# Supplementary material for: Neural response to sad autobiographical recall and sad music listening post recall reveals distinct brain activation in alpha and gamma bands
Source: PLoS One. 2023 Jan 6;18(1):e0279814. doi: 10.1371/journal.pone.0279814 (PMC9821717; doi:10.1371/journal.pone.0279814)
Supplement: S1 Table — 27 ROI defined according to the standard Montreal Neurological Institute (MNI) template and Brodmann areas (BA) in the area of our analysis. (DOCX) [file pone.0279814.s001.docx]

**S1 Table: -**

**Co-ordinates for the 27 regions of interest used in the lag Phase synchronization analysis :-**

**27 ROI defined according to standard Montreal Neurological Institute (MNI) template and Brodmann areas (BA) in the area of our analysis (cingulate cortex complex and PHC) provided in eLoreta software. 13 ROI were present on either side of the hemisphere and one in the central region.**

| S. No. | X-MNI | Y-MNI | Z-MNI | Structure | Brodmann area |
| --- | --- | --- | --- | --- | --- |
| 1 | -5 | -40 | 25 | Posterior Cingulate | Brodmann area 23 |
| 2 | -5 | 0 | 35 | Cingulate Gyrus | Brodmann area 24 |
| 3 | -20 | -35 | -5 | Parahippocampal Gyrus | Brodmann area 27 |
| 4 | -20 | -10 | -25 | Parahippocampal Gyrus | Brodmann area 28 |
| 5 | -5 | -50 | 5 | Posterior Cingulate | Brodmann area 29 |
| 6 | -15 | -60 | 5 | Posterior Cingulate | Brodmann area 30 |
| 7 | -5 | 30 | 20 | Anterior Cingulate | Brodmann area 24 |
| 8 | -5 | 20 | 20 | Anterior Cingulate | Brodmann area 33 |
| 9 | -15 | 0 | -20 | Parahippocampal Gyrus | Brodmann area 34 |
| 10 | -20 | -25 | -20 | Parahippocampal Gyrus | Brodmann area 35 |
| 11 | -30 | -30 | -25 | Parahippocampal Gyrus | Brodmann area 36 |
| 12 | 5 | -45 | 25 | Posterior Cingulate | Brodmann area 23 |
| 13 | 5 | 0 | 35 | Cingulate Gyrus | Brodmann area 24 |
| 14 | 20 | -35 | -5 | Parahippocampal Gyrus | Brodmann area 27 |
| 15 | 20 | -10 | -25 | Parahippocampal Gyrus | Brodmann area 28 |
| 16 | 5 | -50 | 5 | Posterior Cingulate | Brodmann area 29 |
| 17 | 5 | 30 | 20 | Anterior Cingulate | Brodmann area 24 |
| 18 | 0 | 20 | 20 | Anterior Cingulate | Brodmann area 33 |
| 19 | 15 | 0 | -20 | Parahippocampal Gyrus | Brodmann area 34 |
| 20 | 25 | -25 | -20 | Parahippocampal Gyrus | Brodmann area 35 |
| 21 | 30 | -25 | -25 | Parahippocampal Gyrus | Brodmann area 35 |
| 22 | 5 | -40 | 25 | Posterior Cingulate | Brodmann area 23 |
| 23 | 15 | -55 | 5 | Posterior Cingulate | Brodmann area 30 |
| 24 | 5 | 20 | 20 | Anterior Cingulate | Brodmann area 33 |
| 25 | 20 | -25 | -20 | Parahippocampal Gyrus | Brodmann area 35 |
| 26 | -5 | -45 | 25 | Posterior Cingulate | Brodmann area 23 |
| 27 | -25 | -25 | -20 | Parahippocampal Gyrus | Brodmann area 35 |
