# Supplementary material for: Neural response to sad autobiographical recall and sad music listening post recall reveals distinct brain activation in alpha and gamma bands
Source: PLoS One. 2023 Jan 6;18(1):e0279814. doi: 10.1371/journal.pone.0279814 (PMC9821717; doi:10.1371/journal.pone.0279814)
Supplement: S2 Table — 689 Voxels are defined according to the standard Montreal Neurological Institute (MNI) template in our analysis area. (DOCX) [file pone.0279814.s002.docx]

**S2 Table: -**

**Co-ordinates for the 689 regions used in the calculation of current source density (CSD) thus estimating brain activity. Voxels were defined according to standard Montreal Neurological Institute (MNI) template in our analysis area (cingulate cortex complex and PHC) provided in eLoreta software.**

| S. No. | X-MNI | Y-MNI | Z-MNI | Structure | Brodmann area |
| --- | --- | --- | --- | --- | --- |
| 1 | -10 | 35 | -10 | Anterior Cingulate | Brodmann area 32 |
| 2 | -5 | 15 | -10 | Anterior Cingulate | Brodmann area 25 |
| 3 | -5 | 20 | -10 | Anterior Cingulate | Brodmann area 32 |
| 4 | -5 | 25 | -10 | Anterior Cingulate | Brodmann area 32 |
| 5 | -5 | 30 | -10 | Anterior Cingulate | Brodmann area 32 |
| 6 | -5 | 35 | -10 | Anterior Cingulate | Brodmann area 32 |
| 7 | -5 | 40 | -10 | Anterior Cingulate | Brodmann area 32 |
| 8 | 0 | 5 | -10 | Anterior Cingulate | Brodmann area 25 |
| 9 | 0 | 10 | -10 | Anterior Cingulate | Brodmann area 25 |
| 10 | 0 | 15 | -10 | Anterior Cingulate | Brodmann area 25 |
| 11 | 5 | 10 | -10 | Anterior Cingulate | Brodmann area 25 |
| 12 | 5 | 15 | -10 | Anterior Cingulate | Brodmann area 25 |
| 13 | 5 | 20 | -10 | Anterior Cingulate | Brodmann area 32 |
| 14 | 5 | 25 | -10 | Anterior Cingulate | Brodmann area 32 |
| 15 | 5 | 30 | -10 | Anterior Cingulate | Brodmann area 32 |
| 16 | 5 | 35 | -10 | Anterior Cingulate | Brodmann area 32 |
| 17 | 5 | 40 | -10 | Anterior Cingulate | Brodmann area 32 |
| 18 | 10 | 20 | -10 | Anterior Cingulate | Brodmann area 32 |
| 19 | 10 | 35 | -10 | Anterior Cingulate | Brodmann area 32 |
| 20 | -15 | 45 | -5 | Anterior Cingulate | Brodmann area 32 |
| 21 | -10 | 35 | -5 | Anterior Cingulate | Brodmann area 32 |
| 22 | -10 | 45 | -5 | Anterior Cingulate | Brodmann area 32 |
| 23 | -5 | 20 | -5 | Anterior Cingulate | Brodmann area 25 |
| 24 | -5 | 25 | -5 | Anterior Cingulate | Brodmann area 24 |
| 25 | -5 | 30 | -5 | Anterior Cingulate | Brodmann area 24 |
| 26 | -5 | 35 | -5 | Anterior Cingulate | Brodmann area 32 |
| 27 | -5 | 40 | -5 | Anterior Cingulate | Brodmann area 32 |
| 28 | -5 | 45 | -5 | Anterior Cingulate | Brodmann area 32 |
| 29 | 0 | 0 | -5 | Anterior Cingulate | Brodmann area 25 |
| 30 | 0 | 5 | -5 | Anterior Cingulate | Brodmann area 25 |
| 31 | 0 | 10 | -5 | Anterior Cingulate | Brodmann area 25 |
| 32 | 5 | 5 | -5 | Anterior Cingulate | Brodmann area 25 |
| 33 | 5 | 20 | -5 | Anterior Cingulate | Brodmann area 25 |
| 34 | 5 | 25 | -5 | Anterior Cingulate | Brodmann area 24 |
| 35 | 5 | 30 | -5 | Anterior Cingulate | Brodmann area 24 |
| 36 | 5 | 35 | -5 | Anterior Cingulate | Brodmann area 32 |
| 37 | 5 | 40 | -5 | Anterior Cingulate | Brodmann area 32 |
| 38 | 5 | 45 | -5 | Anterior Cingulate | Brodmann area 32 |
| 39 | 10 | 35 | -5 | Anterior Cingulate | Brodmann area 32 |
| 40 | 10 | 45 | -5 | Anterior Cingulate | Brodmann area 32 |
| 41 | 15 | 45 | -5 | Anterior Cingulate | Brodmann area 32 |
| 42 | -15 | 45 | 0 | Anterior Cingulate | Brodmann area 32 |
| 43 | -10 | 45 | 0 | Anterior Cingulate | Brodmann area 32 |
| 44 | -10 | 50 | 0 | Anterior Cingulate | Brodmann area 10 |
| 45 | -5 | 30 | 0 | Anterior Cingulate | Brodmann area 24 |
| 46 | -5 | 35 | 0 | Anterior Cingulate | Brodmann area 32 |
| 47 | -5 | 40 | 0 | Anterior Cingulate | Brodmann area 32 |
| 48 | -5 | 45 | 0 | Anterior Cingulate | Brodmann area 32 |
| 49 | -5 | 50 | 0 | Anterior Cingulate | Brodmann area 32 |
| 50 | 0 | 45 | 0 | Anterior Cingulate | Brodmann area 32 |
| 51 | 0 | 50 | 0 | Anterior Cingulate | Brodmann area 32 |
| 52 | 5 | 30 | 0 | Anterior Cingulate | Brodmann area 24 |
| 53 | 5 | 35 | 0 | Anterior Cingulate | Brodmann area 32 |
| 54 | 5 | 40 | 0 | Anterior Cingulate | Brodmann area 32 |
| 55 | 5 | 45 | 0 | Anterior Cingulate | Brodmann area 32 |
| 56 | 5 | 50 | 0 | Anterior Cingulate | Brodmann area 32 |
| 57 | 5 | 55 | 0 | Anterior Cingulate | Brodmann area 10 |
| 58 | 10 | 45 | 0 | Anterior Cingulate | Brodmann area 32 |
| 59 | 10 | 50 | 0 | Anterior Cingulate | Brodmann area 10 |
| 60 | 15 | 45 | 0 | Anterior Cingulate | Brodmann area 32 |
| 61 | -10 | 45 | 5 | Anterior Cingulate | Brodmann area 32 |
| 62 | -5 | 35 | 5 | Anterior Cingulate | Brodmann area 24 |
| 63 | -5 | 40 | 5 | Anterior Cingulate | Brodmann area 32 |
| 64 | 5 | 35 | 5 | Anterior Cingulate | Brodmann area 24 |
| 65 | 5 | 40 | 5 | Anterior Cingulate | Brodmann area 32 |
| 66 | 10 | 45 | 5 | Anterior Cingulate | Brodmann area 32 |
| 67 | 15 | 45 | 5 | Anterior Cingulate | Brodmann area 32 |
| 68 | -20 | 45 | 10 | Anterior Cingulate | Brodmann area 32 |
| 69 | -15 | 45 | 10 | Anterior Cingulate | Brodmann area 32 |
| 70 | -10 | 45 | 10 | Anterior Cingulate | Brodmann area 32 |
| 71 | -5 | 35 | 10 | Anterior Cingulate | Brodmann area 24 |
| 72 | -5 | 45 | 10 | Anterior Cingulate | Brodmann area 32 |
| 73 | 0 | 35 | 10 | Anterior Cingulate | Brodmann area 24 |
| 74 | 0 | 40 | 10 | Anterior Cingulate | Brodmann area 32 |
| 75 | 0 | 45 | 10 | Anterior Cingulate | Brodmann area 32 |
| 76 | 5 | 35 | 10 | Anterior Cingulate | Brodmann area 24 |
| 77 | 5 | 40 | 10 | Anterior Cingulate | Brodmann area 32 |
| 78 | 5 | 45 | 10 | Anterior Cingulate | Brodmann area 32 |
| 79 | 5 | 50 | 10 | Anterior Cingulate | Brodmann area 10 |
| 80 | 10 | 45 | 10 | Anterior Cingulate | Brodmann area 32 |
| 81 | 15 | 45 | 10 | Anterior Cingulate | Brodmann area 32 |
| 82 | 20 | 45 | 10 | Anterior Cingulate | Brodmann area 32 |
| 83 | -15 | 40 | 15 | Anterior Cingulate | Brodmann area 32 |
| 84 | -10 | 40 | 15 | Anterior Cingulate | Brodmann area 32 |
| 85 | -5 | 25 | 15 | Anterior Cingulate | Brodmann area 24 |
| 86 | -5 | 30 | 15 | Anterior Cingulate | Brodmann area 24 |
| 87 | -5 | 35 | 15 | Anterior Cingulate | Brodmann area 32 |
| 88 | -5 | 40 | 15 | Anterior Cingulate | Brodmann area 32 |
| 89 | -5 | 45 | 15 | Anterior Cingulate | Brodmann area 32 |
| 90 | 0 | 30 | 15 | Anterior Cingulate | Brodmann area 24 |
| 91 | 5 | 25 | 15 | Anterior Cingulate | Brodmann area 24 |
| 92 | 5 | 30 | 15 | Anterior Cingulate | Brodmann area 24 |
| 93 | 5 | 35 | 15 | Anterior Cingulate | Brodmann area 32 |
| 94 | 5 | 40 | 15 | Anterior Cingulate | Brodmann area 32 |
| 95 | 5 | 45 | 15 | Anterior Cingulate | Brodmann area 32 |
| 96 | 10 | 35 | 15 | Anterior Cingulate | Brodmann area 32 |
| 97 | 10 | 40 | 15 | Anterior Cingulate | Brodmann area 32 |
| 98 | 15 | 40 | 15 | Anterior Cingulate | Brodmann area 32 |
| 99 | 20 | 40 | 15 | Anterior Cingulate | Brodmann area 32 |
| 100 | -15 | 35 | 20 | Anterior Cingulate | Brodmann area 32 |
| 101 | -10 | 35 | 20 | Anterior Cingulate | Brodmann area 32 |
| 102 | -5 | 20 | 20 | Anterior Cingulate | Brodmann area 33 |
| 103 | -5 | 25 | 20 | Anterior Cingulate | Brodmann area 24 |
| 104 | -5 | 30 | 20 | Anterior Cingulate | Brodmann area 24 |
| 105 | -5 | 35 | 20 | Anterior Cingulate | Brodmann area 32 |
| 106 | -5 | 40 | 20 | Anterior Cingulate | Brodmann area 32 |
| 107 | 0 | 20 | 20 | Anterior Cingulate | Brodmann area 33 |
| 108 | 0 | 25 | 20 | Anterior Cingulate | Brodmann area 24 |
| 109 | 0 | 30 | 20 | Anterior Cingulate | Brodmann area 24 |
| 110 | 0 | 35 | 20 | Anterior Cingulate | Brodmann area 32 |
| 111 | 5 | 20 | 20 | Anterior Cingulate | Brodmann area 33 |
| 112 | 5 | 25 | 20 | Anterior Cingulate | Brodmann area 24 |
| 113 | 5 | 30 | 20 | Anterior Cingulate | Brodmann area 24 |
| 114 | 5 | 35 | 20 | Anterior Cingulate | Brodmann area 32 |
| 115 | 5 | 40 | 20 | Anterior Cingulate | Brodmann area 32 |
| 116 | 10 | 35 | 20 | Anterior Cingulate | Brodmann area 32 |
| 117 | 15 | 35 | 20 | Anterior Cingulate | Brodmann area 32 |
| 118 | -15 | 35 | 25 | Anterior Cingulate | Brodmann area 32 |
| 119 | -10 | 20 | 25 | Anterior Cingulate | Brodmann area 24 |
| 120 | -10 | 25 | 25 | Anterior Cingulate | Brodmann area 32 |
| 121 | -10 | 35 | 25 | Anterior Cingulate | Brodmann area 32 |
| 122 | -5 | 10 | 25 | Anterior Cingulate | Brodmann area 33 |
| 123 | -5 | 20 | 25 | Anterior Cingulate | Brodmann area 24 |
| 124 | -5 | 25 | 25 | Anterior Cingulate | Brodmann area 24 |
| 125 | -5 | 30 | 25 | Anterior Cingulate | Brodmann area 32 |
| 126 | -5 | 35 | 25 | Anterior Cingulate | Brodmann area 32 |
| 127 | 0 | 20 | 25 | Anterior Cingulate | Brodmann area 33 |
| 128 | 0 | 25 | 25 | Anterior Cingulate | Brodmann area 24 |
| 129 | 0 | 30 | 25 | Anterior Cingulate | Brodmann area 24 |
| 130 | 0 | 35 | 25 | Anterior Cingulate | Brodmann area 32 |
| 131 | 5 | 10 | 25 | Anterior Cingulate | Brodmann area 33 |
| 132 | 5 | 15 | 25 | Anterior Cingulate | Brodmann area 24 |
| 133 | 5 | 20 | 25 | Anterior Cingulate | Brodmann area 24 |
| 134 | 5 | 25 | 25 | Anterior Cingulate | Brodmann area 24 |
| 135 | 5 | 30 | 25 | Anterior Cingulate | Brodmann area 32 |
| 136 | 5 | 35 | 25 | Anterior Cingulate | Brodmann area 32 |
| 137 | 10 | 20 | 25 | Anterior Cingulate | Brodmann area 24 |
| 138 | 10 | 25 | 25 | Anterior Cingulate | Brodmann area 32 |
| 139 | 10 | 35 | 25 | Anterior Cingulate | Brodmann area 32 |
| 140 | 15 | 35 | 25 | Anterior Cingulate | Brodmann area 32 |
| 141 | -10 | 25 | 30 | Anterior Cingulate | Brodmann area 32 |
| 142 | -5 | 25 | 30 | Anterior Cingulate | Brodmann area 24 |
| 143 | -5 | 30 | 30 | Anterior Cingulate | Brodmann area 32 |
| 144 | 10 | 25 | 30 | Anterior Cingulate | Brodmann area 32 |
| 145 | -20 | -45 | 25 | Cingulate Gyrus | Brodmann area 31 |
| 146 | -15 | -45 | 25 | Cingulate Gyrus | Brodmann area 31 |
| 147 | -5 | -60 | 25 | Cingulate Gyrus | Brodmann area 31 |
| 148 | 0 | -60 | 25 | Cingulate Gyrus | Brodmann area 31 |
| 149 | 0 | -55 | 25 | Cingulate Gyrus | Brodmann area 31 |
| 150 | 0 | -45 | 25 | Cingulate Gyrus | Brodmann area 31 |
| 151 | 0 | -40 | 25 | Cingulate Gyrus | Brodmann area 31 |
| 152 | 0 | -35 | 25 | Cingulate Gyrus | Brodmann area 23 |
| 153 | 5 | -60 | 25 | Cingulate Gyrus | Brodmann area 31 |
| 154 | 5 | -55 | 25 | Cingulate Gyrus | Brodmann area 31 |
| 155 | 15 | -45 | 25 | Cingulate Gyrus | Brodmann area 31 |
| 156 | 20 | -45 | 25 | Cingulate Gyrus | Brodmann area 31 |
| 157 | -15 | -45 | 30 | Cingulate Gyrus | Brodmann area 31 |
| 158 | -10 | -45 | 30 | Cingulate Gyrus | Brodmann area 31 |
| 159 | -10 | 15 | 30 | Cingulate Gyrus | Brodmann area 24 |
| 160 | -10 | 30 | 30 | Cingulate Gyrus | Brodmann area 32 |
| 161 | -5 | -60 | 30 | Cingulate Gyrus | Brodmann area 31 |
| 162 | -5 | -45 | 30 | Cingulate Gyrus | Brodmann area 31 |
| 163 | -5 | -40 | 30 | Cingulate Gyrus | Brodmann area 31 |
| 164 | -5 | -35 | 30 | Cingulate Gyrus | Brodmann area 23 |
| 165 | -5 | -30 | 30 | Cingulate Gyrus | Brodmann area 23 |
| 166 | -5 | -25 | 30 | Cingulate Gyrus | Brodmann area 23 |
| 167 | -5 | -20 | 30 | Cingulate Gyrus | Brodmann area 23 |
| 168 | -5 | -15 | 30 | Cingulate Gyrus | Brodmann area 23 |
| 169 | -5 | -10 | 30 | Cingulate Gyrus | Brodmann area 24 |
| 170 | -5 | -5 | 30 | Cingulate Gyrus | Brodmann area 24 |
| 171 | -5 | 0 | 30 | Cingulate Gyrus | Brodmann area 24 |
| 172 | -5 | 10 | 30 | Cingulate Gyrus | Brodmann area 24 |
| 173 | -5 | 15 | 30 | Cingulate Gyrus | Brodmann area 24 |
| 174 | -5 | 20 | 30 | Cingulate Gyrus | Brodmann area 32 |
| 175 | -5 | 35 | 30 | Cingulate Gyrus | Brodmann area 32 |
| 176 | 0 | -45 | 30 | Cingulate Gyrus | Brodmann area 31 |
| 177 | 0 | -40 | 30 | Cingulate Gyrus | Brodmann area 31 |
| 178 | 0 | -35 | 30 | Cingulate Gyrus | Brodmann area 23 |
| 179 | 0 | -30 | 30 | Cingulate Gyrus | Brodmann area 23 |
| 180 | 0 | -25 | 30 | Cingulate Gyrus | Brodmann area 23 |
| 181 | 0 | -20 | 30 | Cingulate Gyrus | Brodmann area 23 |
| 182 | 0 | 15 | 30 | Cingulate Gyrus | Brodmann area 24 |
| 183 | 5 | -45 | 30 | Cingulate Gyrus | Brodmann area 31 |
| 184 | 5 | -40 | 30 | Cingulate Gyrus | Brodmann area 31 |
| 185 | 5 | -35 | 30 | Cingulate Gyrus | Brodmann area 23 |
| 186 | 5 | -30 | 30 | Cingulate Gyrus | Brodmann area 23 |
| 187 | 5 | -25 | 30 | Cingulate Gyrus | Brodmann area 23 |
| 188 | 5 | -20 | 30 | Cingulate Gyrus | Brodmann area 23 |
| 189 | 5 | -15 | 30 | Cingulate Gyrus | Brodmann area 23 |
| 190 | 5 | -10 | 30 | Cingulate Gyrus | Brodmann area 24 |
| 191 | 5 | -5 | 30 | Cingulate Gyrus | Brodmann area 24 |
| 192 | 5 | 0 | 30 | Cingulate Gyrus | Brodmann area 24 |
| 193 | 5 | 5 | 30 | Cingulate Gyrus | Brodmann area 24 |
| 194 | 5 | 10 | 30 | Cingulate Gyrus | Brodmann area 24 |
| 195 | 5 | 15 | 30 | Cingulate Gyrus | Brodmann area 24 |
| 196 | 5 | 20 | 30 | Cingulate Gyrus | Brodmann area 32 |
| 197 | 5 | 25 | 30 | Cingulate Gyrus | Brodmann area 32 |
| 198 | 5 | 30 | 30 | Cingulate Gyrus | Brodmann area 32 |
| 199 | 5 | 35 | 30 | Cingulate Gyrus | Brodmann area 32 |
| 200 | 10 | -45 | 30 | Cingulate Gyrus | Brodmann area 31 |
| 201 | 10 | 15 | 30 | Cingulate Gyrus | Brodmann area 24 |
| 202 | 10 | 20 | 30 | Cingulate Gyrus | Brodmann area 32 |
| 203 | 10 | 30 | 30 | Cingulate Gyrus | Brodmann area 32 |
| 204 | 15 | -45 | 30 | Cingulate Gyrus | Brodmann area 31 |
| 205 | 15 | 30 | 30 | Cingulate Gyrus | Brodmann area 32 |
| 206 | -15 | -45 | 35 | Cingulate Gyrus | Brodmann area 31 |
| 207 | -15 | 15 | 35 | Cingulate Gyrus | Brodmann area 32 |
| 208 | -10 | -45 | 35 | Cingulate Gyrus | Brodmann area 31 |
| 209 | -10 | 10 | 35 | Cingulate Gyrus | Brodmann area 24 |
| 210 | -10 | 15 | 35 | Cingulate Gyrus | Brodmann area 32 |
| 211 | -10 | 25 | 35 | Cingulate Gyrus | Brodmann area 32 |
| 212 | -5 | -45 | 35 | Cingulate Gyrus | Brodmann area 31 |
| 213 | -5 | -20 | 35 | Cingulate Gyrus | Brodmann area 23 |
| 214 | -5 | -15 | 35 | Cingulate Gyrus | Brodmann area 23 |
| 215 | -5 | -10 | 35 | Cingulate Gyrus | Brodmann area 24 |
| 216 | -5 | -5 | 35 | Cingulate Gyrus | Brodmann area 24 |
| 217 | -5 | 0 | 35 | Cingulate Gyrus | Brodmann area 24 |
| 218 | -5 | 5 | 35 | Cingulate Gyrus | Brodmann area 24 |
| 219 | -5 | 10 | 35 | Cingulate Gyrus | Brodmann area 24 |
| 220 | -5 | 15 | 35 | Cingulate Gyrus | Brodmann area 32 |
| 221 | -5 | 25 | 35 | Cingulate Gyrus | Brodmann area 32 |
| 222 | 0 | -45 | 35 | Cingulate Gyrus | Brodmann area 31 |
| 223 | 0 | -40 | 35 | Cingulate Gyrus | Brodmann area 31 |
| 224 | 0 | -35 | 35 | Cingulate Gyrus | Brodmann area 31 |
| 225 | 0 | -30 | 35 | Cingulate Gyrus | Brodmann area 31 |
| 226 | 0 | -25 | 35 | Cingulate Gyrus | Brodmann area 23 |
| 227 | 0 | -20 | 35 | Cingulate Gyrus | Brodmann area 23 |
| 228 | 0 | -15 | 35 | Cingulate Gyrus | Brodmann area 23 |
| 229 | 0 | -10 | 35 | Cingulate Gyrus | Brodmann area 24 |
| 230 | 0 | -5 | 35 | Cingulate Gyrus | Brodmann area 24 |
| 231 | 0 | 0 | 35 | Cingulate Gyrus | Brodmann area 24 |
| 232 | 0 | 10 | 35 | Cingulate Gyrus | Brodmann area 24 |
| 233 | 0 | 15 | 35 | Cingulate Gyrus | Brodmann area 32 |
| 234 | 0 | 20 | 35 | Cingulate Gyrus | Brodmann area 32 |
| 235 | 0 | 25 | 35 | Cingulate Gyrus | Brodmann area 32 |
| 236 | 5 | -45 | 35 | Cingulate Gyrus | Brodmann area 31 |
| 237 | 5 | -40 | 35 | Cingulate Gyrus | Brodmann area 31 |
| 238 | 5 | -35 | 35 | Cingulate Gyrus | Brodmann area 31 |
| 239 | 5 | -30 | 35 | Cingulate Gyrus | Brodmann area 31 |
| 240 | 5 | -25 | 35 | Cingulate Gyrus | Brodmann area 23 |
| 241 | 5 | -20 | 35 | Cingulate Gyrus | Brodmann area 23 |
| 242 | 5 | -15 | 35 | Cingulate Gyrus | Brodmann area 23 |
| 243 | 5 | -10 | 35 | Cingulate Gyrus | Brodmann area 24 |
| 244 | 5 | -5 | 35 | Cingulate Gyrus | Brodmann area 24 |
| 245 | 5 | 0 | 35 | Cingulate Gyrus | Brodmann area 24 |
| 246 | 5 | 5 | 35 | Cingulate Gyrus | Brodmann area 24 |
| 247 | 5 | 10 | 35 | Cingulate Gyrus | Brodmann area 24 |
| 248 | 5 | 15 | 35 | Cingulate Gyrus | Brodmann area 32 |
| 249 | 5 | 20 | 35 | Cingulate Gyrus | Brodmann area 32 |
| 250 | 5 | 25 | 35 | Cingulate Gyrus | Brodmann area 32 |
| 251 | 10 | -45 | 35 | Cingulate Gyrus | Brodmann area 31 |
| 252 | 10 | -10 | 35 | Cingulate Gyrus | Brodmann area 24 |
| 253 | 10 | 10 | 35 | Cingulate Gyrus | Brodmann area 24 |
| 254 | 10 | 15 | 35 | Cingulate Gyrus | Brodmann area 32 |
| 255 | 10 | 25 | 35 | Cingulate Gyrus | Brodmann area 32 |
| 256 | 15 | -45 | 35 | Cingulate Gyrus | Brodmann area 31 |
| 257 | 15 | 15 | 35 | Cingulate Gyrus | Brodmann area 32 |
| 258 | 20 | -45 | 35 | Cingulate Gyrus | Brodmann area 31 |
| 259 | -20 | -35 | 40 | Cingulate Gyrus | Brodmann area 31 |
| 260 | -20 | -30 | 40 | Cingulate Gyrus | Brodmann area 31 |
| 261 | -20 | -20 | 40 | Cingulate Gyrus | Brodmann area 24 |
| 262 | -15 | -35 | 40 | Cingulate Gyrus | Brodmann area 31 |
| 263 | -15 | -30 | 40 | Cingulate Gyrus | Brodmann area 31 |
| 264 | -15 | -25 | 40 | Cingulate Gyrus | Brodmann area 31 |
| 265 | -15 | -20 | 40 | Cingulate Gyrus | Brodmann area 24 |
| 266 | -15 | 0 | 40 | Cingulate Gyrus | Brodmann area 24 |
| 267 | -15 | 5 | 40 | Cingulate Gyrus | Brodmann area 32 |
| 268 | -15 | 10 | 40 | Cingulate Gyrus | Brodmann area 32 |
| 269 | -10 | -50 | 40 | Cingulate Gyrus | Brodmann area 31 |
| 270 | -10 | -45 | 40 | Cingulate Gyrus | Brodmann area 31 |
| 271 | -10 | -30 | 40 | Cingulate Gyrus | Brodmann area 31 |
| 272 | -10 | -25 | 40 | Cingulate Gyrus | Brodmann area 31 |
| 273 | -10 | -20 | 40 | Cingulate Gyrus | Brodmann area 24 |
| 274 | -10 | -10 | 40 | Cingulate Gyrus | Brodmann area 24 |
| 275 | -10 | 0 | 40 | Cingulate Gyrus | Brodmann area 24 |
| 276 | -10 | 5 | 40 | Cingulate Gyrus | Brodmann area 24 |
| 277 | -5 | -50 | 40 | Cingulate Gyrus | Brodmann area 31 |
| 278 | -5 | -45 | 40 | Cingulate Gyrus | Brodmann area 31 |
| 279 | -5 | -40 | 40 | Cingulate Gyrus | Brodmann area 31 |
| 280 | -5 | -35 | 40 | Cingulate Gyrus | Brodmann area 31 |
| 281 | -5 | -30 | 40 | Cingulate Gyrus | Brodmann area 31 |
| 282 | -5 | -20 | 40 | Cingulate Gyrus | Brodmann area 24 |
| 283 | -5 | -15 | 40 | Cingulate Gyrus | Brodmann area 24 |
| 284 | -5 | -10 | 40 | Cingulate Gyrus | Brodmann area 24 |
| 285 | -5 | -5 | 40 | Cingulate Gyrus | Brodmann area 24 |
| 286 | -5 | 0 | 40 | Cingulate Gyrus | Brodmann area 24 |
| 287 | -5 | 5 | 40 | Cingulate Gyrus | Brodmann area 24 |
| 288 | 0 | -35 | 40 | Cingulate Gyrus | Brodmann area 31 |
| 289 | 0 | -30 | 40 | Cingulate Gyrus | Brodmann area 31 |
| 290 | 0 | -25 | 40 | Cingulate Gyrus | Brodmann area 24 |
| 291 | 0 | -20 | 40 | Cingulate Gyrus | Brodmann area 24 |
| 292 | 0 | -15 | 40 | Cingulate Gyrus | Brodmann area 24 |
| 293 | 0 | -10 | 40 | Cingulate Gyrus | Brodmann area 24 |
| 294 | 0 | -5 | 40 | Cingulate Gyrus | Brodmann area 24 |
| 295 | 0 | 0 | 40 | Cingulate Gyrus | Brodmann area 24 |
| 296 | 5 | -50 | 40 | Cingulate Gyrus | Brodmann area 31 |
| 297 | 5 | -45 | 40 | Cingulate Gyrus | Brodmann area 31 |
| 298 | 5 | -40 | 40 | Cingulate Gyrus | Brodmann area 31 |
| 299 | 5 | -35 | 40 | Cingulate Gyrus | Brodmann area 31 |
| 300 | 5 | -30 | 40 | Cingulate Gyrus | Brodmann area 31 |
| 301 | 5 | -25 | 40 | Cingulate Gyrus | Brodmann area 24 |
| 302 | 5 | -20 | 40 | Cingulate Gyrus | Brodmann area 24 |
| 303 | 5 | -15 | 40 | Cingulate Gyrus | Brodmann area 24 |
| 304 | 5 | -10 | 40 | Cingulate Gyrus | Brodmann area 24 |
| 305 | 5 | -5 | 40 | Cingulate Gyrus | Brodmann area 24 |
| 306 | 5 | 0 | 40 | Cingulate Gyrus | Brodmann area 24 |
| 307 | 5 | 5 | 40 | Cingulate Gyrus | Brodmann area 24 |
| 308 | 10 | -50 | 40 | Cingulate Gyrus | Brodmann area 31 |
| 309 | 10 | -45 | 40 | Cingulate Gyrus | Brodmann area 31 |
| 310 | 10 | -30 | 40 | Cingulate Gyrus | Brodmann area 31 |
| 311 | 10 | -20 | 40 | Cingulate Gyrus | Brodmann area 24 |
| 312 | 10 | -15 | 40 | Cingulate Gyrus | Brodmann area 24 |
| 313 | 10 | -10 | 40 | Cingulate Gyrus | Brodmann area 24 |
| 314 | 10 | -5 | 40 | Cingulate Gyrus | Brodmann area 24 |
| 315 | 10 | 0 | 40 | Cingulate Gyrus | Brodmann area 24 |
| 316 | 10 | 5 | 40 | Cingulate Gyrus | Brodmann area 24 |
| 317 | 15 | -45 | 40 | Cingulate Gyrus | Brodmann area 31 |
| 318 | 15 | -35 | 40 | Cingulate Gyrus | Brodmann area 31 |
| 319 | 15 | -30 | 40 | Cingulate Gyrus | Brodmann area 31 |
| 320 | 15 | -25 | 40 | Cingulate Gyrus | Brodmann area 31 |
| 321 | 15 | -20 | 40 | Cingulate Gyrus | Brodmann area 24 |
| 322 | 15 | -15 | 40 | Cingulate Gyrus | Brodmann area 24 |
| 323 | 15 | -10 | 40 | Cingulate Gyrus | Brodmann area 24 |
| 324 | 15 | 0 | 40 | Cingulate Gyrus | Brodmann area 24 |
| 325 | 15 | 5 | 40 | Cingulate Gyrus | Brodmann area 24 |
| 326 | 20 | -35 | 40 | Cingulate Gyrus | Brodmann area 31 |
| 327 | 20 | -30 | 40 | Cingulate Gyrus | Brodmann area 31 |
| 328 | 20 | -25 | 40 | Cingulate Gyrus | Brodmann area 31 |
| 329 | 20 | -20 | 40 | Cingulate Gyrus | Brodmann area 24 |
| 330 | -20 | -35 | 45 | Cingulate Gyrus | Brodmann area 31 |
| 331 | -20 | -30 | 45 | Cingulate Gyrus | Brodmann area 31 |
| 332 | -20 | -20 | 45 | Cingulate Gyrus | Brodmann area 24 |
| 333 | -15 | -35 | 45 | Cingulate Gyrus | Brodmann area 31 |
| 334 | -15 | -30 | 45 | Cingulate Gyrus | Brodmann area 31 |
| 335 | -15 | -25 | 45 | Cingulate Gyrus | Brodmann area 31 |
| 336 | -15 | -20 | 45 | Cingulate Gyrus | Brodmann area 24 |
| 337 | -15 | -5 | 45 | Cingulate Gyrus | Brodmann area 24 |
| 338 | -15 | 0 | 45 | Cingulate Gyrus | Brodmann area 24 |
| 339 | -15 | 5 | 45 | Cingulate Gyrus | Brodmann area 32 |
| 340 | -10 | -30 | 45 | Cingulate Gyrus | Brodmann area 31 |
| 341 | -10 | -25 | 45 | Cingulate Gyrus | Brodmann area 31 |
| 342 | -10 | -20 | 45 | Cingulate Gyrus | Brodmann area 24 |
| 343 | -10 | -15 | 45 | Cingulate Gyrus | Brodmann area 24 |
| 344 | -10 | -10 | 45 | Cingulate Gyrus | Brodmann area 24 |
| 345 | -10 | -5 | 45 | Cingulate Gyrus | Brodmann area 24 |
| 346 | -10 | 0 | 45 | Cingulate Gyrus | Brodmann area 24 |
| 347 | -10 | 5 | 45 | Cingulate Gyrus | Brodmann area 32 |
| 348 | -10 | 15 | 45 | Cingulate Gyrus | Brodmann area 32 |
| 349 | -10 | 20 | 45 | Cingulate Gyrus | Brodmann area 32 |
| 350 | -5 | -40 | 45 | Cingulate Gyrus | Brodmann area 31 |
| 351 | -5 | -30 | 45 | Cingulate Gyrus | Brodmann area 31 |
| 352 | -5 | -20 | 45 | Cingulate Gyrus | Brodmann area 24 |
| 353 | -5 | -15 | 45 | Cingulate Gyrus | Brodmann area 24 |
| 354 | -5 | -10 | 45 | Cingulate Gyrus | Brodmann area 24 |
| 355 | -5 | -5 | 45 | Cingulate Gyrus | Brodmann area 24 |
| 356 | -5 | 0 | 45 | Cingulate Gyrus | Brodmann area 24 |
| 357 | -5 | 5 | 45 | Cingulate Gyrus | Brodmann area 32 |
| 358 | -5 | 10 | 45 | Cingulate Gyrus | Brodmann area 32 |
| 359 | -5 | 15 | 45 | Cingulate Gyrus | Brodmann area 32 |
| 360 | -5 | 20 | 45 | Cingulate Gyrus | Brodmann area 32 |
| 361 | 0 | -5 | 45 | Cingulate Gyrus | Brodmann area 24 |
| 362 | 5 | -20 | 45 | Cingulate Gyrus | Brodmann area 24 |
| 363 | 5 | -15 | 45 | Cingulate Gyrus | Brodmann area 24 |
| 364 | 5 | -10 | 45 | Cingulate Gyrus | Brodmann area 24 |
| 365 | 5 | -5 | 45 | Cingulate Gyrus | Brodmann area 24 |
| 366 | 5 | 0 | 45 | Cingulate Gyrus | Brodmann area 24 |
| 367 | 5 | 5 | 45 | Cingulate Gyrus | Brodmann area 32 |
| 368 | 5 | 10 | 45 | Cingulate Gyrus | Brodmann area 32 |
| 369 | 5 | 15 | 45 | Cingulate Gyrus | Brodmann area 32 |
| 370 | 5 | 20 | 45 | Cingulate Gyrus | Brodmann area 32 |
| 371 | 10 | -30 | 45 | Cingulate Gyrus | Brodmann area 31 |
| 372 | 10 | -20 | 45 | Cingulate Gyrus | Brodmann area 24 |
| 373 | 10 | -15 | 45 | Cingulate Gyrus | Brodmann area 24 |
| 374 | 10 | -10 | 45 | Cingulate Gyrus | Brodmann area 24 |
| 375 | 10 | -5 | 45 | Cingulate Gyrus | Brodmann area 24 |
| 376 | 10 | 0 | 45 | Cingulate Gyrus | Brodmann area 24 |
| 377 | 10 | 5 | 45 | Cingulate Gyrus | Brodmann area 32 |
| 378 | 10 | 15 | 45 | Cingulate Gyrus | Brodmann area 32 |
| 379 | 10 | 20 | 45 | Cingulate Gyrus | Brodmann area 32 |
| 380 | 15 | -35 | 45 | Cingulate Gyrus | Brodmann area 31 |
| 381 | 15 | -30 | 45 | Cingulate Gyrus | Brodmann area 31 |
| 382 | 15 | -25 | 45 | Cingulate Gyrus | Brodmann area 31 |
| 383 | 15 | -20 | 45 | Cingulate Gyrus | Brodmann area 24 |
| 384 | 15 | -10 | 45 | Cingulate Gyrus | Brodmann area 24 |
| 385 | 15 | -5 | 45 | Cingulate Gyrus | Brodmann area 24 |
| 386 | 15 | 0 | 45 | Cingulate Gyrus | Brodmann area 24 |
| 387 | 15 | 5 | 45 | Cingulate Gyrus | Brodmann area 32 |
| 388 | 20 | -35 | 45 | Cingulate Gyrus | Brodmann area 31 |
| 389 | 20 | -30 | 45 | Cingulate Gyrus | Brodmann area 31 |
| 390 | 20 | -25 | 45 | Cingulate Gyrus | Brodmann area 31 |
| 391 | 20 | -20 | 45 | Cingulate Gyrus | Brodmann area 24 |
| 392 | -20 | 5 | 50 | Cingulate Gyrus | Brodmann area 32 |
| 393 | -15 | -10 | 50 | Cingulate Gyrus | Brodmann area 24 |
| 394 | -15 | -5 | 50 | Cingulate Gyrus | Brodmann area 24 |
| 395 | -15 | 5 | 50 | Cingulate Gyrus | Brodmann area 24 |
| 396 | -10 | -10 | 50 | Cingulate Gyrus | Brodmann area 24 |
| 397 | -10 | -5 | 50 | Cingulate Gyrus | Brodmann area 24 |
| 398 | -10 | 5 | 50 | Cingulate Gyrus | Brodmann area 24 |
| 399 | -5 | -10 | 50 | Cingulate Gyrus | Brodmann area 24 |
| 400 | -5 | -5 | 50 | Cingulate Gyrus | Brodmann area 24 |
| 401 | -5 | 0 | 50 | Cingulate Gyrus | Brodmann area 24 |
| 402 | 0 | -5 | 50 | Cingulate Gyrus | Brodmann area 24 |
| 403 | 0 | 0 | 50 | Cingulate Gyrus | Brodmann area 24 |
| 404 | 5 | -10 | 50 | Cingulate Gyrus | Brodmann area 24 |
| 405 | 5 | -5 | 50 | Cingulate Gyrus | Brodmann area 24 |
| 406 | 5 | 0 | 50 | Cingulate Gyrus | Brodmann area 24 |
| 407 | 10 | -10 | 50 | Cingulate Gyrus | Brodmann area 24 |
| 408 | 10 | -5 | 50 | Cingulate Gyrus | Brodmann area 24 |
| 409 | 10 | 5 | 50 | Cingulate Gyrus | Brodmann area 24 |
| 410 | 15 | -10 | 50 | Cingulate Gyrus | Brodmann area 24 |
| 411 | 15 | -5 | 50 | Cingulate Gyrus | Brodmann area 24 |
| 412 | 15 | 5 | 50 | Cingulate Gyrus | Brodmann area 24 |
| 413 | 20 | 5 | 50 | Cingulate Gyrus | Brodmann area 24 |
| 414 | -35 | -25 | -30 | Parahippocampal Gyrus | Brodmann area 36 |
| 415 | -35 | -20 | -30 | Parahippocampal Gyrus | Brodmann area 36 |
| 416 | -30 | -25 | -30 | Parahippocampal Gyrus | Brodmann area 36 |
| 417 | -30 | -20 | -30 | Parahippocampal Gyrus | Brodmann area 36 |
| 418 | -25 | -20 | -30 | Parahippocampal Gyrus | Brodmann area 36 |
| 419 | -25 | -15 | -30 | Parahippocampal Gyrus | Brodmann area 35 |
| 420 | -20 | -10 | -30 | Parahippocampal Gyrus | Brodmann area 35 |
| 421 | 20 | -10 | -30 | Parahippocampal Gyrus | Brodmann area 35 |
| 422 | 25 | -20 | -30 | Parahippocampal Gyrus | Brodmann area 36 |
| 423 | 25 | -15 | -30 | Parahippocampal Gyrus | Brodmann area 35 |
| 424 | 30 | -25 | -30 | Parahippocampal Gyrus | Brodmann area 36 |
| 425 | 30 | -20 | -30 | Parahippocampal Gyrus | Brodmann area 36 |
| 426 | 30 | -15 | -30 | Parahippocampal Gyrus | Brodmann area 36 |
| 427 | 35 | -25 | -30 | Parahippocampal Gyrus | Brodmann area 36 |
| 428 | 35 | -20 | -30 | Parahippocampal Gyrus | Brodmann area 36 |
| 429 | -40 | -30 | -25 | Parahippocampal Gyrus | Brodmann area 36 |
| 430 | -35 | -30 | -25 | Parahippocampal Gyrus | Brodmann area 36 |
| 431 | -30 | -30 | -25 | Parahippocampal Gyrus | Brodmann area 36 |
| 432 | -30 | -25 | -25 | Parahippocampal Gyrus | Brodmann area 35 |
| 433 | -25 | -25 | -25 | Parahippocampal Gyrus | Brodmann area 35 |
| 434 | -25 | -20 | -25 | Parahippocampal Gyrus | Brodmann area 35 |
| 435 | -20 | -20 | -25 | Parahippocampal Gyrus | Brodmann area 28 |
| 436 | -20 | -15 | -25 | Parahippocampal Gyrus | Brodmann area 28 |
| 437 | -20 | -10 | -25 | Parahippocampal Gyrus | Brodmann area 28 |
| 438 | 20 | -15 | -25 | Parahippocampal Gyrus | Brodmann area 28 |
| 439 | 20 | -10 | -25 | Parahippocampal Gyrus | Brodmann area 28 |
| 440 | 25 | -25 | -25 | Parahippocampal Gyrus | Brodmann area 35 |
| 441 | 25 | -20 | -25 | Parahippocampal Gyrus | Brodmann area 35 |
| 442 | 30 | -30 | -25 | Parahippocampal Gyrus | Brodmann area 35 |
| 443 | 30 | -25 | -25 | Parahippocampal Gyrus | Brodmann area 35 |
| 444 | 35 | -30 | -25 | Parahippocampal Gyrus | Brodmann area 36 |
| 445 | 40 | -30 | -25 | Parahippocampal Gyrus | Brodmann area 36 |
| 446 | -35 | -30 | -20 | Parahippocampal Gyrus | Brodmann area 36 |
| 447 | -35 | -25 | -20 | Parahippocampal Gyrus | Brodmann area 36 |
| 448 | -30 | -30 | -20 | Parahippocampal Gyrus | Brodmann area 36 |
| 449 | -30 | 5 | -20 | Parahippocampal Gyrus | Brodmann area 34 |
| 450 | -25 | -35 | -20 | Parahippocampal Gyrus | Brodmann area 36 |
| 451 | -25 | -30 | -20 | Parahippocampal Gyrus | Brodmann area 36 |
| 452 | -25 | -25 | -20 | Parahippocampal Gyrus | Brodmann area 35 |
| 453 | -25 | 5 | -20 | Parahippocampal Gyrus | Brodmann area 34 |
| 454 | -20 | -30 | -20 | Parahippocampal Gyrus | Brodmann area 35 |
| 455 | -20 | -25 | -20 | Parahippocampal Gyrus | Brodmann area 35 |
| 456 | -20 | -20 | -20 | Parahippocampal Gyrus | Brodmann area 28 |
| 457 | -20 | -15 | -20 | Parahippocampal Gyrus | Brodmann area 28 |
| 458 | -20 | -10 | -20 | Parahippocampal Gyrus | Brodmann area 34 |
| 459 | -20 | 5 | -20 | Parahippocampal Gyrus | Brodmann area 34 |
| 460 | -15 | -10 | -20 | Parahippocampal Gyrus | Brodmann area 34 |
| 461 | -15 | -5 | -20 | Parahippocampal Gyrus | Brodmann area 34 |
| 462 | -15 | 0 | -20 | Parahippocampal Gyrus | Brodmann area 34 |
| 463 | 15 | -10 | -20 | Parahippocampal Gyrus | Brodmann area 34 |
| 464 | 15 | -5 | -20 | Parahippocampal Gyrus | Brodmann area 34 |
| 465 | 15 | 0 | -20 | Parahippocampal Gyrus | Brodmann area 34 |
| 466 | 20 | -30 | -20 | Parahippocampal Gyrus | Brodmann area 35 |
| 467 | 20 | -25 | -20 | Parahippocampal Gyrus | Brodmann area 35 |
| 468 | 20 | -20 | -20 | Parahippocampal Gyrus | Brodmann area 28 |
| 469 | 20 | -15 | -20 | Parahippocampal Gyrus | Brodmann area 28 |
| 470 | 20 | -10 | -20 | Parahippocampal Gyrus | Brodmann area 34 |
| 471 | 20 | 5 | -20 | Parahippocampal Gyrus | Brodmann area 34 |
| 472 | 25 | -35 | -20 | Parahippocampal Gyrus | Brodmann area 36 |
| 473 | 25 | -30 | -20 | Parahippocampal Gyrus | Brodmann area 36 |
| 474 | 25 | -25 | -20 | Parahippocampal Gyrus | Brodmann area 35 |
| 475 | 25 | 5 | -20 | Parahippocampal Gyrus | Brodmann area 34 |
| 476 | 30 | -30 | -20 | Parahippocampal Gyrus | Brodmann area 36 |
| 477 | 30 | 5 | -20 | Parahippocampal Gyrus | Brodmann area 34 |
| 478 | 35 | -30 | -20 | Parahippocampal Gyrus | Brodmann area 36 |
| 479 | 40 | -25 | -20 | Parahippocampal Gyrus | Brodmann area 20 |
| 480 | -40 | -30 | -15 | Parahippocampal Gyrus | Brodmann area 36 |
| 481 | -35 | -35 | -15 | Parahippocampal Gyrus | Brodmann area 36 |
| 482 | -35 | -30 | -15 | Parahippocampal Gyrus | Brodmann area 36 |
| 483 | -30 | -45 | -15 | Parahippocampal Gyrus | Brodmann area 37 |
| 484 | -30 | -40 | -15 | Parahippocampal Gyrus | Brodmann area 37 |
| 485 | -25 | -45 | -15 | Parahippocampal Gyrus | Brodmann area 37 |
| 486 | -25 | -40 | -15 | Parahippocampal Gyrus | Brodmann area 36 |
| 487 | -25 | -35 | -15 | Parahippocampal Gyrus | Brodmann area 36 |
| 488 | -25 | -30 | -15 | Parahippocampal Gyrus | Brodmann area 36 |
| 489 | -20 | -40 | -15 | Parahippocampal Gyrus | Brodmann area 36 |
| 490 | -20 | -35 | -15 | Parahippocampal Gyrus | Brodmann area 35 |
| 491 | -20 | -30 | -15 | Parahippocampal Gyrus | Brodmann area 35 |
| 492 | -20 | -25 | -15 | Parahippocampal Gyrus | Brodmann area 35 |
| 493 | -20 | -20 | -15 | Parahippocampal Gyrus | Brodmann area 35 |
| 494 | -20 | -15 | -15 | Parahippocampal Gyrus | Brodmann area 28 |
| 495 | -20 | 0 | -15 | Parahippocampal Gyrus | Brodmann area 34 |
| 496 | -15 | -10 | -15 | Parahippocampal Gyrus | Brodmann area 28 |
| 497 | -15 | -5 | -15 | Parahippocampal Gyrus | Brodmann area 28 |
| 498 | -15 | 0 | -15 | Parahippocampal Gyrus | Brodmann area 34 |
| 499 | 15 | -10 | -15 | Parahippocampal Gyrus | Brodmann area 28 |
| 500 | 15 | -5 | -15 | Parahippocampal Gyrus | Brodmann area 28 |
| 501 | 15 | 0 | -15 | Parahippocampal Gyrus | Brodmann area 34 |
| 502 | 20 | -40 | -15 | Parahippocampal Gyrus | Brodmann area 36 |
| 503 | 20 | -35 | -15 | Parahippocampal Gyrus | Brodmann area 35 |
| 504 | 20 | -30 | -15 | Parahippocampal Gyrus | Brodmann area 35 |
| 505 | 20 | -25 | -15 | Parahippocampal Gyrus | Brodmann area 35 |
| 506 | 20 | -20 | -15 | Parahippocampal Gyrus | Brodmann area 35 |
| 507 | 20 | -15 | -15 | Parahippocampal Gyrus | Brodmann area 28 |
| 508 | 20 | 0 | -15 | Parahippocampal Gyrus | Brodmann area 34 |
| 509 | 25 | -45 | -15 | Parahippocampal Gyrus | Brodmann area 37 |
| 510 | 25 | -40 | -15 | Parahippocampal Gyrus | Brodmann area 36 |
| 511 | 25 | -35 | -15 | Parahippocampal Gyrus | Brodmann area 36 |
| 512 | 25 | -30 | -15 | Parahippocampal Gyrus | Brodmann area 36 |
| 513 | 30 | -45 | -15 | Parahippocampal Gyrus | Brodmann area 37 |
| 514 | 30 | -35 | -15 | Parahippocampal Gyrus | Brodmann area 36 |
| 515 | 35 | -35 | -15 | Parahippocampal Gyrus | Brodmann area 36 |
| 516 | 40 | -30 | -15 | Parahippocampal Gyrus | Brodmann area 36 |
| 517 | -35 | -45 | -10 | Parahippocampal Gyrus | Brodmann area 19 |
| 518 | -30 | -50 | -10 | Parahippocampal Gyrus | Brodmann area 19 |
| 519 | -30 | -45 | -10 | Parahippocampal Gyrus | Brodmann area 19 |
| 520 | -30 | -40 | -10 | Parahippocampal Gyrus | Brodmann area 37 |
| 521 | -25 | -55 | -10 | Parahippocampal Gyrus | Brodmann area 19 |
| 522 | -25 | -50 | -10 | Parahippocampal Gyrus | Brodmann area 19 |
| 523 | -25 | -45 | -10 | Parahippocampal Gyrus | Brodmann area 37 |
| 524 | -25 | -40 | -10 | Parahippocampal Gyrus | Brodmann area 36 |
| 525 | -25 | -30 | -10 | Parahippocampal Gyrus | Brodmann area 27 |
| 526 | -25 | -20 | -10 | Parahippocampal Gyrus | Brodmann area 28 |
| 527 | -20 | -55 | -10 | Parahippocampal Gyrus | Brodmann area 19 |
| 528 | -20 | -50 | -10 | Parahippocampal Gyrus | Brodmann area 19 |
| 529 | -20 | -45 | -10 | Parahippocampal Gyrus | Brodmann area 19 |
| 530 | -20 | -40 | -10 | Parahippocampal Gyrus | Brodmann area 36 |
| 531 | -20 | -35 | -10 | Parahippocampal Gyrus | Brodmann area 35 |
| 532 | -20 | -30 | -10 | Parahippocampal Gyrus | Brodmann area 28 |
| 533 | -20 | -25 | -10 | Parahippocampal Gyrus | Brodmann area 28 |
| 534 | -15 | -40 | -10 | Parahippocampal Gyrus | Brodmann area 30 |
| 535 | -15 | -35 | -10 | Parahippocampal Gyrus | Brodmann area 30 |
| 536 | 15 | -40 | -10 | Parahippocampal Gyrus | Brodmann area 30 |
| 537 | 15 | -35 | -10 | Parahippocampal Gyrus | Brodmann area 30 |
| 538 | 20 | -55 | -10 | Parahippocampal Gyrus | Brodmann area 19 |
| 539 | 20 | -50 | -10 | Parahippocampal Gyrus | Brodmann area 19 |
| 540 | 20 | -45 | -10 | Parahippocampal Gyrus | Brodmann area 19 |
| 541 | 20 | -40 | -10 | Parahippocampal Gyrus | Brodmann area 36 |
| 542 | 20 | -35 | -10 | Parahippocampal Gyrus | Brodmann area 35 |
| 543 | 20 | -30 | -10 | Parahippocampal Gyrus | Brodmann area 28 |
| 544 | 20 | -25 | -10 | Parahippocampal Gyrus | Brodmann area 28 |
| 545 | 25 | -55 | -10 | Parahippocampal Gyrus | Brodmann area 19 |
| 546 | 25 | -50 | -10 | Parahippocampal Gyrus | Brodmann area 19 |
| 547 | 25 | -45 | -10 | Parahippocampal Gyrus | Brodmann area 37 |
| 548 | 25 | -40 | -10 | Parahippocampal Gyrus | Brodmann area 36 |
| 549 | 25 | -30 | -10 | Parahippocampal Gyrus | Brodmann area 27 |
| 550 | 25 | -20 | -10 | Parahippocampal Gyrus | Brodmann area 28 |
| 551 | 30 | -50 | -10 | Parahippocampal Gyrus | Brodmann area 19 |
| 552 | 30 | -45 | -10 | Parahippocampal Gyrus | Brodmann area 19 |
| 553 | 30 | -40 | -10 | Parahippocampal Gyrus | Brodmann area 36 |
| 554 | 35 | -45 | -10 | Parahippocampal Gyrus | Brodmann area 19 |
| 555 | 35 | -40 | -10 | Parahippocampal Gyrus | Brodmann area 37 |
| 556 | -35 | -45 | -5 | Parahippocampal Gyrus | Brodmann area 19 |
| 557 | -30 | -50 | -5 | Parahippocampal Gyrus | Brodmann area 19 |
| 558 | -30 | -45 | -5 | Parahippocampal Gyrus | Brodmann area 19 |
| 559 | -25 | -55 | -5 | Parahippocampal Gyrus | Brodmann area 19 |
| 560 | -25 | -50 | -5 | Parahippocampal Gyrus | Brodmann area 19 |
| 561 | -25 | -30 | -5 | Parahippocampal Gyrus | Brodmann area 27 |
| 562 | -20 | -55 | -5 | Parahippocampal Gyrus | Brodmann area 19 |
| 563 | -20 | -50 | -5 | Parahippocampal Gyrus | Brodmann area 19 |
| 564 | -20 | -45 | -5 | Parahippocampal Gyrus | Brodmann area 19 |
| 565 | -20 | -35 | -5 | Parahippocampal Gyrus | Brodmann area 27 |
| 566 | -20 | -30 | -5 | Parahippocampal Gyrus | Brodmann area 27 |
| 567 | -15 | -40 | -5 | Parahippocampal Gyrus | Brodmann area 30 |
| 568 | -15 | -35 | -5 | Parahippocampal Gyrus | Brodmann area 27 |
| 569 | -10 | -45 | -5 | Parahippocampal Gyrus | Brodmann area 30 |
| 570 | -10 | -40 | -5 | Parahippocampal Gyrus | Brodmann area 30 |
| 571 | 10 | -40 | -5 | Parahippocampal Gyrus | Brodmann area 30 |
| 572 | 10 | -35 | -5 | Parahippocampal Gyrus | Brodmann area 27 |
| 573 | 15 | -40 | -5 | Parahippocampal Gyrus | Brodmann area 30 |
| 574 | 15 | -35 | -5 | Parahippocampal Gyrus | Brodmann area 27 |
| 575 | 20 | -55 | -5 | Parahippocampal Gyrus | Brodmann area 19 |
| 576 | 20 | -50 | -5 | Parahippocampal Gyrus | Brodmann area 19 |
| 577 | 20 | -45 | -5 | Parahippocampal Gyrus | Brodmann area 19 |
| 578 | 20 | -35 | -5 | Parahippocampal Gyrus | Brodmann area 27 |
| 579 | 20 | -30 | -5 | Parahippocampal Gyrus | Brodmann area 27 |
| 580 | 25 | -55 | -5 | Parahippocampal Gyrus | Brodmann area 19 |
| 581 | 25 | -50 | -5 | Parahippocampal Gyrus | Brodmann area 19 |
| 582 | 25 | -35 | -5 | Parahippocampal Gyrus | Brodmann area 27 |
| 583 | 25 | -30 | -5 | Parahippocampal Gyrus | Brodmann area 27 |
| 584 | 30 | -50 | -5 | Parahippocampal Gyrus | Brodmann area 19 |
| 585 | 30 | -45 | -5 | Parahippocampal Gyrus | Brodmann area 19 |
| 586 | 35 | -45 | -5 | Parahippocampal Gyrus | Brodmann area 19 |
| 587 | -25 | -55 | 0 | Parahippocampal Gyrus | Brodmann area 30 |
| 588 | -15 | -45 | 0 | Parahippocampal Gyrus | Brodmann area 30 |
| 589 | -15 | -35 | 0 | Parahippocampal Gyrus | Brodmann area 27 |
| 590 | -10 | -50 | 0 | Parahippocampal Gyrus | Brodmann area 30 |
| 591 | -10 | -45 | 0 | Parahippocampal Gyrus | Brodmann area 30 |
| 592 | -10 | -40 | 0 | Parahippocampal Gyrus | Brodmann area 30 |
| 593 | -10 | -35 | 0 | Parahippocampal Gyrus | Brodmann area 27 |
| 594 | 10 | -50 | 0 | Parahippocampal Gyrus | Brodmann area 30 |
| 595 | 10 | -45 | 0 | Parahippocampal Gyrus | Brodmann area 30 |
| 596 | 10 | -40 | 0 | Parahippocampal Gyrus | Brodmann area 30 |
| 597 | 10 | -35 | 0 | Parahippocampal Gyrus | Brodmann area 27 |
| 598 | 15 | -45 | 0 | Parahippocampal Gyrus | Brodmann area 30 |
| 599 | 15 | -35 | 0 | Parahippocampal Gyrus | Brodmann area 27 |
| 600 | 20 | -50 | 0 | Parahippocampal Gyrus | Brodmann area 30 |
| 601 | 25 | -55 | 0 | Parahippocampal Gyrus | Brodmann area 30 |
| 602 | -25 | -70 | 5 | Posterior Cingulate | Brodmann area 30 |
| 603 | -20 | -70 | 5 | Posterior Cingulate | Brodmann area 30 |
| 604 | -20 | -65 | 5 | Posterior Cingulate | Brodmann area 30 |
| 605 | -20 | -60 | 5 | Posterior Cingulate | Brodmann area 30 |
| 606 | -15 | -60 | 5 | Posterior Cingulate | Brodmann area 30 |
| 607 | -15 | -55 | 5 | Posterior Cingulate | Brodmann area 30 |
| 608 | -10 | -55 | 5 | Posterior Cingulate | Brodmann area 30 |
| 609 | -10 | -50 | 5 | Posterior Cingulate | Brodmann area 29 |
| 610 | -10 | -45 | 5 | Posterior Cingulate | Brodmann area 29 |
| 611 | -5 | -60 | 5 | Posterior Cingulate | Brodmann area 30 |
| 612 | -5 | -55 | 5 | Posterior Cingulate | Brodmann area 30 |
| 613 | -5 | -50 | 5 | Posterior Cingulate | Brodmann area 29 |
| 614 | 5 | -60 | 5 | Posterior Cingulate | Brodmann area 30 |
| 615 | 5 | -55 | 5 | Posterior Cingulate | Brodmann area 30 |
| 616 | 5 | -50 | 5 | Posterior Cingulate | Brodmann area 29 |
| 617 | 10 | -45 | 5 | Posterior Cingulate | Brodmann area 29 |
| 618 | 15 | -55 | 5 | Posterior Cingulate | Brodmann area 30 |
| 619 | 20 | -65 | 5 | Posterior Cingulate | Brodmann area 30 |
| 620 | 20 | -60 | 5 | Posterior Cingulate | Brodmann area 30 |
| 621 | 20 | -55 | 5 | Posterior Cingulate | Brodmann area 30 |
| 622 | 25 | -70 | 5 | Posterior Cingulate | Brodmann area 30 |
| 623 | 25 | -65 | 5 | Posterior Cingulate | Brodmann area 30 |
| 624 | -25 | -70 | 10 | Posterior Cingulate | Brodmann area 30 |
| 625 | -20 | -65 | 10 | Posterior Cingulate | Brodmann area 30 |
| 626 | -20 | -60 | 10 | Posterior Cingulate | Brodmann area 30 |
| 627 | -15 | -65 | 10 | Posterior Cingulate | Brodmann area 30 |
| 628 | -15 | -60 | 10 | Posterior Cingulate | Brodmann area 30 |
| 629 | -10 | -70 | 10 | Posterior Cingulate | Brodmann area 30 |
| 630 | -10 | -65 | 10 | Posterior Cingulate | Brodmann area 30 |
| 631 | -5 | -70 | 10 | Posterior Cingulate | Brodmann area 30 |
| 632 | -5 | -65 | 10 | Posterior Cingulate | Brodmann area 30 |
| 633 | -5 | -60 | 10 | Posterior Cingulate | Brodmann area 30 |
| 634 | -5 | -55 | 10 | Posterior Cingulate | Brodmann area 29 |
| 635 | -5 | -50 | 10 | Posterior Cingulate | Brodmann area 29 |
| 636 | 5 | -70 | 10 | Posterior Cingulate | Brodmann area 30 |
| 637 | 5 | -65 | 10 | Posterior Cingulate | Brodmann area 30 |
| 638 | 5 | -60 | 10 | Posterior Cingulate | Brodmann area 30 |
| 639 | 5 | -55 | 10 | Posterior Cingulate | Brodmann area 29 |
| 640 | 5 | -50 | 10 | Posterior Cingulate | Brodmann area 29 |
| 641 | 10 | -70 | 10 | Posterior Cingulate | Brodmann area 30 |
| 642 | 10 | -65 | 10 | Posterior Cingulate | Brodmann area 30 |
| 643 | 15 | -65 | 10 | Posterior Cingulate | Brodmann area 30 |
| 644 | 15 | -60 | 10 | Posterior Cingulate | Brodmann area 30 |
| 645 | 20 | -70 | 10 | Posterior Cingulate | Brodmann area 30 |
| 646 | 20 | -65 | 10 | Posterior Cingulate | Brodmann area 30 |
| 647 | 20 | -60 | 10 | Posterior Cingulate | Brodmann area 30 |
| 648 | 25 | -70 | 10 | Posterior Cingulate | Brodmann area 30 |
| 649 | -25 | -70 | 15 | Posterior Cingulate | Brodmann area 18 |
| 650 | -20 | -65 | 15 | Posterior Cingulate | Brodmann area 31 |
| 651 | -10 | -70 | 15 | Posterior Cingulate | Brodmann area 31 |
| 652 | -5 | -70 | 15 | Posterior Cingulate | Brodmann area 31 |
| 653 | -5 | -60 | 15 | Posterior Cingulate | Brodmann area 23 |
| 654 | -5 | -55 | 15 | Posterior Cingulate | Brodmann area 30 |
| 655 | -5 | -50 | 15 | Posterior Cingulate | Brodmann area 30 |
| 656 | 0 | -65 | 15 | Posterior Cingulate | Brodmann area 23 |
| 657 | 0 | -60 | 15 | Posterior Cingulate | Brodmann area 23 |
| 658 | 0 | -55 | 15 | Posterior Cingulate | Brodmann area 23 |
| 659 | 0 | -50 | 15 | Posterior Cingulate | Brodmann area 30 |
| 660 | 5 | -70 | 15 | Posterior Cingulate | Brodmann area 31 |
| 661 | 5 | -65 | 15 | Posterior Cingulate | Brodmann area 23 |
| 662 | 5 | -60 | 15 | Posterior Cingulate | Brodmann area 23 |
| 663 | 5 | -55 | 15 | Posterior Cingulate | Brodmann area 23 |
| 664 | 5 | -50 | 15 | Posterior Cingulate | Brodmann area 30 |
| 665 | 10 | -70 | 15 | Posterior Cingulate | Brodmann area 31 |
| 666 | 10 | -55 | 15 | Posterior Cingulate | Brodmann area 23 |
| 667 | 20 | -65 | 15 | Posterior Cingulate | Brodmann area 31 |
| 668 | -5 | -60 | 20 | Posterior Cingulate | Brodmann area 31 |
| 669 | -5 | -55 | 20 | Posterior Cingulate | Brodmann area 31 |
| 670 | -5 | -50 | 20 | Posterior Cingulate | Brodmann area 30 |
| 671 | 0 | -55 | 20 | Posterior Cingulate | Brodmann area 23 |
| 672 | 0 | -50 | 20 | Posterior Cingulate | Brodmann area 30 |
| 673 | 0 | -45 | 20 | Posterior Cingulate | Brodmann area 30 |
| 674 | 5 | -60 | 20 | Posterior Cingulate | Brodmann area 23 |
| 675 | 5 | -55 | 20 | Posterior Cingulate | Brodmann area 23 |
| 676 | 5 | -50 | 20 | Posterior Cingulate | Brodmann area 30 |
| 677 | 5 | -45 | 20 | Posterior Cingulate | Brodmann area 30 |
| 678 | 10 | -60 | 20 | Posterior Cingulate | Brodmann area 31 |
| 679 | -5 | -55 | 25 | Posterior Cingulate | Brodmann area 31 |
| 680 | -5 | -45 | 25 | Posterior Cingulate | Brodmann area 23 |
| 681 | -5 | -40 | 25 | Posterior Cingulate | Brodmann area 23 |
| 682 | -5 | -35 | 25 | Posterior Cingulate | Brodmann area 23 |
| 683 | -5 | -30 | 25 | Posterior Cingulate | Brodmann area 23 |
| 684 | 0 | -50 | 25 | Posterior Cingulate | Brodmann area 23 |
| 685 | 5 | -50 | 25 | Posterior Cingulate | Brodmann area 23 |
| 686 | 5 | -45 | 25 | Posterior Cingulate | Brodmann area 23 |
| 687 | 5 | -40 | 25 | Posterior Cingulate | Brodmann area 23 |
| 688 | 5 | -35 | 25 | Posterior Cingulate | Brodmann area 23 |
| 689 | 5 | -30 | 25 | Posterior Cingulate | Brodmann area 23 |
