# Supplementary material for: Neural response to sad autobiographical recall and sad music listening post recall reveals distinct brain activation in alpha and gamma bands
Source: PLoS One. 2023 Jan 6;18(1):e0279814. doi: 10.1371/journal.pone.0279814 (PMC9821717; doi:10.1371/journal.pone.0279814)
Supplement: S3 Table — Statistical strength analysis of the experiment. (DOCX) [file pone.0279814.s003.docx]

**S3 Table: -**

**The detailed table of the power analysis of the experiment highlighting the statistical strength (power) and effect size is presented below. All the results of eLoreta and behaviour analysis are shown in absolute value for the ease of comparison.**

| **Fig 1** | | | | |
| --- | --- | --- | --- | --- |
| **Ms Vs SAR (alpha1)** | | | | |
| **No: of Significant connections** | **α- value** | **t -value** | **effect size** | **power value** |
| 14 | 0.0167 | 4.6339 to 5.2226 | 1.1965 to 1.3485 | 0.95 to 0.98 |
| **Hemisphere localization : Left** | 0.05 | 14.6426 | 3.7807 | 0.99 |
|  |  |  |  |  |
| **Ms Vs SAR (alpha2)** | | | | |
| **No: of Significant connections** | **α- value** | **t -value** | **effect size** | **power value** |
| 108 | 0.0167 | 4.6322 to 7.5765 | 1.1960 to 1.9562 | 0.95 to 0.99 |
| **Hemisphere localization : Right** | 0.05 | 25.8362 | 6.6709 | 0.99 |
|  |  |  |  |  |
| **Ms Vs SAR (gamma)** | | | | |
| **No: of Significant connections** | **α- value** | **t -value** | **effect size** | **power value** |
| 8 | 0.0167 | 4.638 to 5.8001 | 1.1975 to 1.4976 | 0.95 to 0.99 |
| **Hemisphere localization : No** | 0.05 | 1 | 0.2582 | 0.154 |
| **Fig 2** | | | | |
|  |  |  | **SAR Vs BL (alpha1)** |  |
| **No: of Significant connections** | **α- value** | **t -value** | **effect size** | **power value** |
| 1 | 0.0167 | 4.6681 | 1.2053 | 0.96 |
| **Hemisphere localization : No** | 0.05 | 0 | 0 | 0 |
|  |  |  |  |  |
|  |  |  | **SAR Vs BL (alpha2)** |  |
| **No: of Significant connections** | **α- value** | **t -value** | **effect size** | **power value** |
| 111 | 0.0167 | 4.6652 to 6.7256 | 1.2045 to 1.7365 | 0.96 to 0.99 |
| **Hemisphere localization : Right** | 0.05 | 10.0111 | 2.5849 | 0.99 |
|  |  |  |  |  |
|  |  |  | **SAR Vs BL (gamma)** |  |
| **No: of Significant connections** | **α- value** | **t -value** | **effect size** | **power value** |
| 17 | 0.0167 | 4.6497 to 6.0957 | 1.2005 to 1.5739 | 0.96 to 0.99 |
| **Hemisphere localization : Right** | 0.05 | 8.211 | 2.1201 | 0.99 |
| **Fig 3** | | | | |
| **Ms Vs SAR(alpha2)** | | | | |
| **No: of Significant connections** | **α- value** | **t -value** | **effect size** | **power value** |
| 287 | 0.0167 | 3.9700 to 5.4927 | 1.0251 to 1.4182 | 0.87 to 0.99 |
| **Hemisphere localization : Right** | 0.05 | 4.3752 | 1.1297 | 0.98 |
|  |  |  |  |  |
| **Ms Vs SAR(gamma)** | | | | |
| **No: of Significant connections** | **α- value** | **t -value** | **effect size** | **power value** |
| 103 | 0.0167 | 3.9935 to 6.4373 | -1.0311 to -1.6621 | 0.88 to 0.99 |
| **Hemisphere localization : Right** | 0.05 | 31.134 | 8.0388 | 0.99 |
|  |  |  |  |  |
| **Ms Vs BL(alpha2)** | | | | |
| **No: of Significant connections** | **α- value** | **t -value** | **effect size** | **power value** |
| 8 | 0.0167 | 3.6114 to 4.7791 | 0.9325 to 1.234 | 0.80 to 0.96 |
| **Hemisphere localization : Left** | 0.05 | 13.5695 | 3.5036 | 0.99 |
| **Fig 4** | | | | |
| **SAR VS BL(alpha2)** | | | | |
| **No: of Significant connections** | **α- value** | **t -value** | **effect size** | **power value** |
| 191 | 0.0167 | 4.0654 to 5.7798 | 1.0497 to -1.4923 | 0.89 to 0.99 |
| **Hemisphere localization : Right** | 0.05 | 24.4616 | 6.316 | 0.99 |
|  |  |  |  |  |
| **SAR VS BL(gamma)** | | | | |
| **No: of Significant connections** | **α- value** | **t -value** | **effect size** | **power value** |
| 105 | 0.0167 | 4.0697 to 6.1474 | 1.0508 to 1.5873 | 0.89 to 0.99 |
| **Hemisphere localization : Right** | 0.05 | 245.9086 | 63.69 | 0.99 |
|  |  |  |  |  |
|  | **Fig 5** | | | |
|  | **BL Vs SAR** | | | |
|  | **α- value** | **t -value** | **effect size** | **power value** |
|  | 0.0167 | 8.663 | 2.236 | 0.99 |
|  |  |  |  |  |
|  | **BL Vs Ms** | | | |
|  | **α- value** | **t -value** | **effect size** | **power value** |
|  | 0.0167 | -6.094 | 1.5735 | 0.99 |
