# Supplementary material for: Neural response to sad autobiographical recall and sad music listening post recall reveals distinct brain activation in alpha and gamma bands
Source: PLoS One. 2023 Jan 6;18(1):e0279814. doi: 10.1371/journal.pone.0279814 (PMC9821717; doi:10.1371/journal.pone.0279814)
Supplement: Attachment — Submitted filename: Response_To_Reviewers.docx [file pone.0279814.s004.docx]

PONE-D-21-33402

Neural response to Sad Autobiographical recall and Sad Music listening post recall reveals distinct brain activation in alpha and gamma bands.

PLOS ONE

Dear Editor, board members, and Esteemed Reviewers,

The authors would like to convey their sincere gratitude to the reviewers for taking out time to review this manuscript.

Thank you for the valuable comments, feedback, and recommendations, which offered us an opportunity to improve the quality of the manuscript.

We have sincerely taken into account all the comments and have tried our best to address all comments from reviewers. We have also revised the manuscript accordingly.

We have highlighted our response to the comments in **blue** colour.

Reviewer #1: Review for PONE_21-33402

Title: Neural response to Sad Autobiographical recall and Sad Music listening post recall
reveals distinct brain activation in alpha and gamma bands.

General Overview:

Summarise main findings:

Overall, this is an innovative study, which was conceptualised, designed, and will certainly add to the literature. The study explored the EEG source strength, connectivity and changes associated with sad autobiographical recall and listening to sad music. In particular, alpha band lag phase-synchronization during sad music listening, especially within and between the Posterior cingulate cortex (PCC) and (PHC) when compared to sad autobiographical recall. The main feature found was that there may be two distinct for sad music listening and sad recall. The effects were found in alpha and gamma band.

Limitations and Strengths:

Overall, this is an innovative study, and well-conceived. However, there may be a few minor issues requiring clarification and restructuring of the narrative. For example:

• Why not other EEG frequencies, like theta? Beta? They are discussed I the introduction in the context of emotional memory. The authors need to explain why significance was not seen for these bands.

We thank the reviewer for the comments. We have duly noted the reviewer’s remark and have incorporated a relevant text in the manuscript in the **Discussion section** regarding theta and beta bands

(page 12, line 457-459)

We do observe the difference (for α < 0.05) in mean lag-phase synchronization analysis in the theta and Beta2 bands, but the difference did not reach a significant level with Bonferroni correction) (for α < 0.05/ 15)

(page 7, line 270-271)

Table 1: Mean Phase coherence analysis.

| **Bands/ Condns.** | **Mean: Ms** | **Mean: SAR** | **Mean: BL** | **Ms Vs SAR**  **p-value:** | **Ms Vs BL**  **p-value:** | **SAR Vs BL**  **p-value:** |
| --- | --- | --- | --- | --- | --- | --- |
| Theta | 0.025817149 | 0.03172679 | 0.020874407 | 0.118866655 | 0.196483555 | 0.03562922 |
| Alpha1 | 0.120878241 | 0.046470902 | 0.122535169 | 0.000593192* | 0.906776823 | 0.001469557* |
| Alpha2 | 0.167790108 | 0.051265335 | 0.182173521 | 5.09E-05* | 0.478311389 | 7.39E-05* |
| Beta2 | 0.032824653 | 0.021487982 | 0.028102876 | 0.024355147 | 0.214273223 | 0.123957443 |
| Gamma | 0.015758674 | 0.026168399 | 0.014148663 | 0.001372256* | 0.624069884 | 0.01196064 |

Method section (page 6, line 234-235)

Post hoc pairwise comparison revealed a significant difference only in alpha1, alpha2, and gamma bands which were used for further analysis.

• Laterality could be associated with positive and negative emotion (Ahern et al 1985). Some disorders like PTSD may have triggers associated with sad music; perhaps the authors may need to highlight this early.

Thank you for the comments. We have edited the Introduction section based upon the reviewer’s feedback.

(page 3, line 70-77)

The Valance lateralization hypothesis postulates that the left hemisphere is specialized in processing positive emotions, and the right hemisphere is specialized in processing negative emotions [^[1]^]. A recent review favoured the Right hemisphere dominance hypothesis that emotions, irrespective of the valance, are primarily processed in the right hemisphere [1]. Lateralization studies for memories have also found right hemisphere dominance during negative autobiography memory retrieval [^[2]^], [^[3]^], [^[4]^], [^[5]^]. Since our experiment involves negative valanced emotions and memories, we expect the results to have right hemisphere dominance.

(page 2, line 28-31)

A review on the usage of sad music concluded that although psychologically healthy individuals tend to be benefited from sad music listening during adverse circumstances, the authors cautioned that the usage of sad music among individuals with a history of depression, PTSD, etc [^[6]^] could be maladaptive.

• The research may be culturally sensitive and will need further research in other cultures.

Yes, the current study maintains the cultural salience of the music. However, studies from other cultures would further validate it. We have added the following in the discussion section

(page 14, line 554-556)

Sad music is used as a coping strategy across several cultures. The present results give a promising hypothesis to validate and extend the findings to other cultures and practices.

• Perhaps suggest reorganising format so Methods appears before results. There are some explanations in Methods which assist with the interpretation of the Results.

We thank the reviewer for the feedback. We have reorganised the manuscript accordingly.

• Why not also have an additional 20 female participants?

Thank you for the comment.

Men and women have been reported to use dissimilar brain networks for processing sad, depressing, and fun musical stimuli [^[7]^]. The difference is especially prominent for negative valanced stimulus [7]. The present work recruited only male participants due to practical limitations. Expanding the scope by recruiting additional female participants is not possible at this stage. However, we will keep this suggestion in mind when planning future studies

The study is unique and will certainly add to the literature and be hypothesis generating especially for other clinical and cultural scenarios.

Abstract
• A good summary of the conceptual framework, methodologies, results, and outcomes. Could be more concise and clear.

Thank you for the comments. We have edited the abstract section accordingly.

Introduction
• Concepts are generally well introduced. Good rationale presented early in the narrative.
• However, the first three paragraphs could be more concise, seems like the same concepts are being covered again. Need to make it clear from a behavioral, social and biological point of view.

We thank the reviewer for the feedback. We have edited and reorganised the introduction section accordingly.

• The research may be culturally sensitive and will need further research in other cultures. Is it relevant to other cultures?

Thank you for the comment.

The current study maintains the cultural salience of the music and the participants. We agree that a further research in other cultures might be useful.

We have added the following in the discussion section (page 14, line 554-556)

Since Sad music is used as a coping strategy across several culture. The present results give a promising hypothesis to validate and extend the findings to other cultures and practices.

•Should also address emotional regulation in the context of the salience network as well.

Thank you for the suggestion. We have added the following section in our discussion (page 14, line 560-569).

Salience network plays a vital role in social and affective tasks involving emotions and introspections [^[8]^]. Salience network compromises of anterior insula, dorsal anterior cingulate cortex, and three key subcortical structures: amygdala, ventral striatum, and substantia nigra/ventral tegmental area [^[9]^]. Our results do support the benefit of listening to music in terms of emotion regulation and enhanced alertness. However, although EEG analysis is favoured with high temporal resolution, nevertheless it lacks spatial resolution. Future studies through fMRI would give better spatial resolution and expand the research by quantifying the involvement of cortical and sub cortical regions viz salience network in emotional coping through sad music listening.

• The final paragraph clarifies the rationale and outcomes, however the term ”probing tool” may not be an accurate description for the methodologies used. Reference to beta but no data presented in Figures later.

Thank you for the feedback.

We have replaced the “probing tool” with the “investigating tool”. We have added the relevant data for theta and Beta band analysis in the table 1

(page 7, line 270).

| **Bands/ Condns.** | **Mean: Ms** | **Mean: SAR** | **Mean: BL** | **Ms Vs SAR**  **p-value:** | **Ms Vs BL**  **p-value:** | **SAR Vs BL**  **p-value:** |
| --- | --- | --- | --- | --- | --- | --- |
| Theta | 0.025817149 | 0.03172679 | 0.020874407 | 0.118866655 | 0.196483555 | 0.03562922 |
| Alpha1 | 0.120878241 | 0.046470902 | 0.122535169 | 0.000593192* | 0.906776823 | 0.001469557* |
| Alpha2 | 0.167790108 | 0.051265335 | 0.182173521 | 5.09E-05* | 0.478311389 | 7.39E-05* |
| Beta2 | 0.032824653 | 0.021487982 | 0.028102876 | 0.024355147 | 0.214273223 | 0.123957443 |
| Gamma | 0.015758674 | 0.026168399 | 0.014148663 | 0.001372256* | 0.624069884 | 0.01196064 |

However, since the result did not reach a significant level with Bonferroni correction for theta and Beta bands, further analysis and figure depictions were not done.

• May require some restructuring to improve flow of the narrative and rationale.

We thank the reviewer for the feedback. We have edited and reorganised the relevant sections accordingly.

• Overall, well referenced and conceptual design supported.

Results
• Well-constructed overall

• Some of the methodology described in Results, should be covered in Methods, for example, little mention of functional coherence analysis in Methods.

We thank the reviewer for the feedback. We have moved the relevant section from Results to Methods

Page 6, line 209-215. (Text moved to method section).

Brain connectivity was computed by measuring lagged phase synchronization through eLoreta at the source level between all pairs of 27 ROI resulting in 729 connections.27 ROI were defined according to the standard Montreal Neurological Institute (MNI) template and Brodmann areas (BA) in our area of analysis (cingulate cortex complex and PHC). 13 ROI were present on either side of the hemisphere and one in the central region (for the complete co-ordinates of ROI, see S1 Table).

We added further relevant descriptions in the method sections for functional coherence analysis. Details are mentioned below in the method section (bullet point number 2).

• Line 152-153 for the first time indicates that only the 3 bands reported significance across conditions. It may be advisable to indicate this in Methods as well.

Thank you for the comment. We have added the following description in the method section under Statistical analysis subsection

(page 6, line 234-235)

Post hoc pairwise comparison revealed a significant difference only in alpha1, alpha2, and gamma bands which were used for further analysis.

• Need to refer to Tables in narrative eg (See Table 1).

Thank you for the feedback. We have referred the table at the appropriate place in the manuscript.

Discussion

• A well-constructed narrative, however, some proof reading and restructure necessary to improve flow of the narrative would be useful.
• Avoid the use of the term “probing tool”, investigative tool may be more appropriate.

Thank you for the feedback. We have edited and restructured the discussion section accordingly.

We have replaced the “probing tool” with the “investigating tool”.

• Some large paragraphs may require some rewriting to improve flow of the narrative. Starting line 283

Thank you for the comment. We have edited the section to make it more clear and better flow of the narrative.

• Some repetition

Thank you for the feedback. We have edited the section and removed any irrelevant or repetitive information.

• Line 398 “In summary..”, should be a short paragraph and not followed by new and additional narrative. “In summary..”, again at line 459

Thank for the feedback. We edited the section to make it clearer, concise and removed any irrelevant or repetitive information.

• A limitations narrative is very brief and could have been expanded.

We thank the reviewer for the comment. We have incorporated relevant text in the discussion section as below (page 14, line 548-572)

However, the current model needs to be tested with different kinds of sad music, and mood inductions procedures. The present study involved only male participants. Studies suggest dissimilar networks being used by males and females [7], thus limiting the generalizability of the findings.

We need to check whether listening to sad music can further enhance the internally oriented process when the mood induction procedure is similar (internally oriented). The internally oriented mood induction method would help to investigate wider brain regions. Sad music is used as a coping strategy across several cultures. The present results give a promising hypothesis to validate and extend the findings to other cultures and practices. We wonder whether greater left-sided symmetry observed during enhanced functional connectivity in alpha1 band is associated with the positive/approach emotion aspect of listening to sad music compared to SAR state. Further studies are required to ascertain it.

The Salience network plays a vital role in social and affective tasks involving emotions and introspections [8]. The Salience network compromises of anterior insula, dorsal anterior cingulate cortex, and three key subcortical structures: amygdala, ventral striatum, and substantia nigra/ventral tegmental area [9]. Our results do support the benefit of listening to music in terms of emotion regulation and enhanced alertness. However, although EEG analysis is favoured with the high temporal resolution, nevertheless it lacks spatial resolution. Future studies through fMRI would give better spatial resolution and expand the research by quantifying the involvement of cortical and sub-cortical regions viz salience network in emotional coping through sad music listening. Although neural correlates difference between sad music listening and SAR gives potential mechanisms responsible for the self-regulatory goals, further studies are needed to establish the causal connections between the observed effect and sad music excerpt.

Methods

• This section is well constructed overall; however, subheadings may have been useful in clarifying the protocols etc

Thank you for the suggestion. We have restructured the method section accordingly.

• An appropriate methodology for functional connectivity was selected and applied. However, more information about why, is required.

We thank the reviewer for the valuable feedback. We have the following section in the Method under subsection

Source connectivity Analysis (page 6, line 200-215)

Brain regions coordinate with one another to accomplish any cognitive or perceptual tasks. Functional connectivity between two brain regions during the task gives an index for how well these regions synchronized to accomplish the task [^[10]^]. Information flow between two regions is directly proportional to functional connectivity between them [10]. Thus, functional connectivity is a good instrument to quantify how well the regions are connected in a particular task/condition. We selected functional connectivity as our first investigating tool.

We chose lag phase synchronization for it measures non-linear functional connectivity independent of power fluctuation, any instantaneous zero lagged component, volume conduction and is resistant to non-physiological artifacts, particularly low spatial resolution [^[11]^].

Brain connectivity was computed by measuring lagged phase synchronization through eLoreta at source level between all pairs of 27 ROI resulting in 729 connections. 27 ROI were defined according to the standard Montreal Neurological Institute (MNI) template and Brodmann areas (BA) in our area of analysis (cingulate cortex complex and PHC). 13 ROI were present on either side of the hemisphere and one in the central region (for the complete co-ordinates of ROI, see S1 Table).

• Why do authors cite eLORETA paper? Did they use this technique? Or was it sLORETA alone? This needs to be clarified. eLORETA is not the same as sLORETA and there may be some confusion in the interpretation of data.

We thank the reviewer for bringing notice to this point. We used eLORETA alone for this study. We have corrected our typo mistake in the manuscript accordingly.

• Eye, blink and muscle movement may have contributed to EEG changes during SAR. How was this controlled for and was the minimize movement enough? Could this be achieved post analysis, not just visual inspection? Should reference the algorithms used in EEGLAB toolbox.

We thank the reviewer for the comment.

The Sad autobiographical recall task was conducted by carefully keeping the artifacts from eye and muscle movements to the minimum possible level:

All the participants were requested to sit as still as possible while simultaneously performing the task in a condition similar to a natural situation. Participants sat in an adjustable chair in front of a table. A pillow was used at the back to release any tension during the experiment. All the participants were instructed to keep their gaze fixed on the cross printed
centrally on a blank paper kept over the table during baseline and sad music listening
conditions. The cross sheet was replaced with a writing pad upon the table during SAR.

They were asked to support their elbow while writing to reduce the hand movements. Post recording, the data was visually checked for eye, muscle, or electrode movement artifacts. Subsequently, these bad electrodes were interpolated in EEG LAB.

However, to further eliminate the artifacts, in addition to visual inspection and interpolation of the bad channels, we applied independent component analysis (ICA) and SASICA (Semi-Automatic Selection of Independent Component Analysis) [^[12]^].

ICA-based algorithms are quite successful in cleaning the EEG data of eyes and muscles [^[13]^]. SASICA helps in cleaning of the eye and muscle artifacts based upon several algorithms in addition to SASICA, like FASTER (Fully Automated Statistical Thresholding for EEG artifacts Rejection) [^[14]^], ADJUST (Automatic EEG artifacts Detection based on the Joint Use of Spatial and Temporal features) [^[15]^]

Simultaneous analysis through different algorithms makes SASICA a promising tool for artifacts correction, SASICA has been used in several earlier studies [^[16]^], [^[17]^].

Following quantitative measures as calculated by SASICA were used for eliminating artifacts:

Eye artifacts and Blink: ADJUST used temporal Kurtosis, spatial average difference, spatial variance difference measures for eye blink correction. And maximum epoch variance, spatial eye difference measures for eye artifacts correction. We recorded 4 EOG in addition to 32 EEG channels. SASICA and FASTER used correlational analysis to further clean eye blink, and eye artifacts.

Muscles artifacts: High Median Gradient & Hurst Exponent measures calculated through FASTER, Generic discontinuity spatial feature measure calculated through ADJUST, and Low autocorrelation of time-course measure calculated through SASICA were used in eliminating muscles artifacts.

We have added the relevant text and reference of the software in the method section (sub section: EEG recording and preprocessing) of the manuscript at: page 5, line 174-185.

• What was the final number of participants tested once some were removed for previous exposure to music, artifacts and after the KPD scale?

The final number of participants tested were 15 after removing those with previous exposure to the music, artifacts and after the KPD scale.

• Brand of amplifier/supplier ref?

We used a g.HIamp bio-signal amplifier (Guger Technologies, OG, Graz, Austria) to capture the EEG from the participants. We have added the changes in the method section of the manuscript (page 5 line 162)

• Why was an earlobe reference used? What about linked earlobes? This would contribute to laterality shifts.

We recorded our EEG reading with the left earlobe as a reference. However, Referencing is a linear transformation of data and data can be re-reference offline. Thus, to have no laterality bias/shifts, we re-referenced our EEG data to Average referencing based on Makoto's pre-processing pipeline [^[18]^] using EEGLAB. We followed Makoto's pre-processing pipeline for data pre-processing. All our EEG analysis was done on average referenced data which takes care of laterality issue.

• Was the baseline resting state eyes open or closed?

Baseline resting state was with eye open.

All the participants were instructed to sit calmly with their gaze fixed on the cross printed
centrally on a blank paper kept over the table during baseline and sad music listening
conditions. The cross sheet was replaced with a writing pad upon the table during SAR.

• Do the authors find any relationships with other bands, seeing that had these data.

We also observed the difference in mean lag-phase synchronization analysis in the theta and Beta2 bands, but the difference did not reach a significant level with Bonferroni correction. Post hoc pairwise comparison revealed a significant difference only in alpha1, alpha2, and gamma bands which were used for further analysis. Thus, we observed a strong relationship in alpha1, alpha2 and gamma bands and a weak relationship for theta and Beta bands.

• More details about how the sLORETA data was calculated and statistically analysed.

We thank the reviewer for the feedback. We edited the method section according as below

Subsection: EEG Source Analysis (page 5, line 187-193 and 195-198)

Exact Low Resolution Electromagnetic Tomography (eLORETA) is a weighted minimal norm inverse method and performs a three-dimensional, discrete and linear source localization [^[19]^]. It is uniquely equipped with the ability to exactly localize a test point source. Source localization and analysis of 32 channel EEG data was conducted through eLoreta. Intracortical current source density (CSD) was calculated as a linear weighted sum of potentials at scalp through eLoreta software at 6239 voxels sampled at 5 mm resolution. CSD was calculated as a linear weighted sum of potentials at the scalp through eLORETA software. The following frequency bands were analysed: delta (1–4 Hz); theta (4.5–8 Hz); alpha1 (8.5–10 Hz); alpha2 (10.5–12 Hz); beta1 (12.5–18 Hz); beta2 (18.5–21 Hz); beta3 (21.5–30 Hz); gamma (35-44Hz). Brain activity was analysed across 689 voxels related to CC, PCC, ACC, and PHC in our region of interest. 689 Voxels are defined according to the standard Montreal Neurological Institute (MNI) template in our analysis area.

Subsection: Statistical Analysis (page 6, line 230-244)

A two-tailed t-test with alpha value of 0.05 was used to compare the mean values and analyse subjective assessments. Repeated ANOVA was used to investigate simple and interaction effects between bands and conditions for the mean value of EEG data. Bonferroni correction was performed for multiple testing across conditions (Baseline, SAR, Music). Post hoc pairwise comparison revealed a significant difference only in alpha1, alpha2, and gamma bands which were used for further analysis. We used SPSS toolbox for statistical analysis. Statistical analysis for eLoreta source level data at the voxels level is characterized by multiple testing problems. We used non-parametric mapping method (SnPM) via randomization statistical tests (threshold set at 5% probability, Fisher’s permutation method) as implemented in eLoreta statistical package to address the multiple correction problem for testing across all voxels, connections and bands [^[20]^]. eLoreta uses 5000 randomizations to determine critical probability threshold values with correction for multiple comparisons across voxels and frequencies. SnPm method in Loreta analysis has been validated in several earlier studies [^[21]^].

• A power analysis may have been useful in highlighting the statistical strength of effects and sLORETA data. Is n <20 sufficient for significance?

We thank the author for the valuable feedback.

We have done the experiment’s power analysis highlighting the statistical strength (power) and effect size. We have submitted the detailed table in the supplementary document along with the manuscript (S3 Table).

All the results from eLoreta analysis show t – value within the range from 3.6114 to 7.5765 with effect size ranging from 0.9325 to 1.9562, and power value ranging from 0.80 to 0.99. Results also show high statistical strength (power) for hemispheric lateralization as well as behavioural analysis. Power analysis for all the results shows the values above 0.80, thereby supporting the findings obtained in the experiment. Table below shows the power analysis for the study.

| **Fig 1** | | | | |
| --- | --- | --- | --- | --- |
| **Ms Vs SAR (alpha1)** | | | | |
| **No: of Significant connections** | **α- value** | **t -value** | **effect size** | **power value** |
| 14 | 0.0167 | 4.6339 to 5.2226 | 1.1965 to 1.3485 | 0.95 to 0.98 |
| **Hemisphere localization: Left hemisphere significant difference** | 0.05 | 14.6426 | 3.7807 | 0.99 |
| **Ms Vs SAR (alpha2)** | | | | |
| **No: of Significant connections** | **α- value** | **t -value** | **effect size** | **power value** |
| 108 | 0.0167 | 4.6322 to 7.5765 | 1.1960 to 1.9562 | 0.95 to 0.99 |
| **Hemisphere localization: Right hemisphere significant difference** | 0.05 | 25.8362 | 6.6709 | 0.99 |
| **Ms Vs SAR (gamma)** | | | | |
| **No: of Significant connections** | **α- value** | **t -value** | **effect size** | **power value** |
| 8 | 0.0167 | 4.638 to 5.8001 | 1.1975 to 1.4976 | 0.95 to 0.99 |
| **Hemisphere localization:**  **No significant difference** | 0.05 | 1 | 0.2582 | 0.154 |
| **Fig 2** | | | | |
| **SAR Vs BL (alpha1)** | | | | |
| **No: of Significant connections** | **α- value** | **t -value** | **effect size** | **power value** |
| 1 | 0.0167 | 4.6681 | 1.2053 | 0.96 |
| **Hemisphere localization:**  **No significant difference** | 0.05 | 0 | 0 | 0 |
| **SAR Vs BL (alpha2)** | | | | |
| **No: of Significant connections** | **α- value** | **t -value** | **effect size** | **power value** |
| 111 | 0.0167 | 4.6652 to 6.7256 | 1.2045 to 1.7365 | 0.96 to 0.99 |
| **Hemisphere localization: Right**  **hemisphere significant difference** | 0.05 | 10.0111 | 2.5849 | 0.99 |
| **SAR Vs BL (gamma)** | | | | |
| **No: of Significant connections** | **α- value** | **t -value** | **effect size** | **power value** |
| 17 | 0.0167 | 4.6497 to 6.0957 | 1.2005 to 1.5739 | 0.96 to 0.99 |
| **Hemisphere localization: Right**  **hemisphere significant difference** | 0.05 | 8.211 | 2.1201 | 0.99 |
| **Fig 3** | | | | |
| **Ms Vs SAR (alpha2)** | | | | |
| **No: of Significant connections** | **α- value** | **t -value** | **effect size** | **power value** |
| 287 | 0.0167 | 3.9700 to 5.4927 | 1.0251 to 1.4182 | 0.87 to 0.99 |
| **Hemisphere localization: Right hemisphere significant difference** | 0.05 | 4.3752 | 1.1297 | 0.98 |
| **Ms Vs SAR (gamma)** | | | | |
| **No: of Significant connections** | **α- value** | **t -value** | **effect size** | **power value** |
| 103 | 0.0167 | 3.9935 to 6.4373 | 1.0311 to 1.6621 | 0.88 to 0.99 |
| **Hemisphere localization: Right hemisphere significant difference** | 0.05 | 31.134 | 8.0388 | 0.99 |
| **Ms Vs BL (alpha2)** | | | | |
| **No: of Significant connections** | **α- value** | **t -value** | **effect size** | **power value** |
| 8 | 0.0167 | 3.6114 to 4.7791 | 0.9325 to 1.234 | 80 to 96.95 |
| **Hemisphere localization: Left hemisphere significant difference** | 0.05 | 13.5695 | 3.5036 | 0.99 |
| **Fig 4** | | | | |
| **SAR VS BL (alpha2)** | | | | |
| **No: of Significant connections** | **α- value** | **t -value** | **effect size** | **power value** |
| 191 | 0.0167 | 4.0654 to 5.7798 | 1.0497 to 1.4923 | 0.89 to 0.99 |
| **Hemisphere localization: Right hemisphere significant difference** | 0.05 | 24.4616 | 6.316 | 0.99 |
| **SAR VS BL (gamma)** | | | | |
| **No: of Significant connections** | **α- value** | **t -value** | **effect size** | **power value** |
| 105 | 0.0167 | 4.0697 to 6.1474 | 1.0508 to 1.5873 | 0.89 to 0.99 |
| **Hemisphere localization: Right hemisphere significant difference** | 0.05 | 245.9086 | 63.69 | 0.99 |
|  | **Fig 5** | | | |
|  | **BL Vs SAR** | | | |
|  | **α- value** | **t -value** | **effect size** | **power value** |
|  | 0.0167 | 8.663 | 2.236 | 0.99 |
|  | **BL Vs Ms** | | | |
|  | **α- value** | **t -value** | **effect size** | **power value** |
|  | 0.0167 | 6.094 | 1.5735 | 0.99 |

• Some of the methodology described in Results, should be covered in Methods, for example, little mention of functional coherence analysis in Methods.

We thank the reviewer for the feedback. We have edited the method section accordingly.

Please refer method section bullet point number 2.

• What platform was used to calculate the statistics? SPSS, Toolbox?

We used SPSS toolbox for statistical analysis.

• Why was phase coherence selected as a tool?

We thank the reviewer for the feedback. We have edited the method section and added the relevant text. Please refer method section bullet point number 2.

References:
• Excellent review of the field with most primary references used.

Figure Legends:
• Most images are well constructed and easy to follow.

Reviewer #2: Manuscript ID: PONE-D-21-33402

Title: Neural response to sad autobiographical recall and sad music listening post recall reveals distinct brain activation in alpha and gamma bands.

Authors: Ashish Gupta, Braj Bhushan and Laxmidhar Behera


In this research, Gupta and colleagues compared EEG rhythms collected at rest (baseline) with those induced by sad autobiographical recall (SAR) and upon exposure to sad music. Results focused in particular on sad music listening condition and revealed a significant involvement of Alpha and Gamma EEG bands in specific region of interest (ROIs) including CC, ACC, PCC and PHC. Authors concludes that the SAR condition induced enhanced gamma-band activity, suggesting increased content binding capacity, whereas the sad music listening was associated with an enhanced alpha band activity, suggesting increased content-specific information processing.
The paper addresses an important topic. However, there are important issues in the methods and data discussion that significantly affect the quality of the manuscript.

First of all, I’m puzzled about the authors’ choice over the frequency range used to define the delta EEG rhythm (i.e., 1.5-6 Hz): usually, delta waves range between 0/0.5/1 Hz to 4 Hz, whereas 4-6 Hz represent the lower frequencies of theta rhythm (full range = 4-8 Hz). I recommend to revise the analyses on delta and theta bands adjusting the frequency ranges, in line with most of qEEG literature.

We thank the reviewer for giving the valuable feedback. We revised the analysis after adjusting the frequency ranges and updated the manuscript accordingly. The updated sections are as follows:

Method section (page 5, line 193-195)

The following frequency bands were analysed delta (1–4 Hz); theta (4.5–8 Hz); alpha1 (8.5–10 Hz); alpha2 (10.5–12 Hz); beta1 (12.5–18 Hz); beta2 (18.5–21 Hz); beta3 (21.5–30 Hz); gamma (35-44Hz).

Result Section (page 7, line 251-256)

We found a significant interaction effect with a Greenhouse-Geisser correction between bands and conditions (F_3.785,52.991_ = 15.303, p < 0.001). One-way repeated measure ANOVA was performed to study the simple effect of bands. Results show significant effect of bands on functional connectivity for theta band (F_2,28_ = 3.711, p=0.037), alpha1 (F_2,28_ = 13.328, p<0.001), alpha2 (F_2,28_ = 22.710, p< 0.001), beta2 (F_2,28_ = 3.915, p= 0.032), and gamma (F_2,28_ = 7.402, p= 0.003) bands.

The second, critical issue regards results discussion and interpretation. All the discussion focused on the increased phase synchronization/connectivity in the Alpha (1 and 2) band during sad music listening vs. SAR condition, and authors interpreted these findings as “an overall enhancement in the brain’s processing in areas related to memory and spatial/action information region specifically, in addition to emotion”. I’m not convinced this is the correct interpretation, as alpha rhythm is physiologically associated with cortical inhibition (see, for example, Klimesch, W. (2012). Alpha-band oscillations, attention, and controlled access to stored information. Trends in Cognitive Sciences 16, 606-617). In this perspective, increased alpha connectivity should be interpreted as increased inhibition of these regions in the brain during sad music listening, or, from the other perspective, a decreased inhibition of the memory/spatial-action information/emotion circuit during SAR condition. This interpretation is further supported by the lack of “any significant difference between the functional connectivity between sad music listening condition and baseline resting-state for alpha band”.
Whether sad music listening induces “an overall enhancement in the brain’s processing in areas related to memory and spatial/action information region specifically, in addition to emotion”, this may be found also with respect to resting state (baseline) condition.
In addition, the decreased connectivity in alpha band (suggesting decreased inhibition within the brain network) found in SAR vs. sad music listening condition matches the result found in gamma EEG band, as “[…] Detailed brain activity analysis in the gamma band shows significantly enhanced activity during SAR state compared to sad music listening AND BASELINE RESTING STATE […] Gamma activity is a robust signature of episodic memory.”
I therefore recommend that the authors reconsider their interpretations of alpha rhythm in a more coherent discussion, in line with the suggested evidence.

We thank the reviewer for their valuable feedback.

There are several ways to define neural synchronization. The two most prominent types of synchronization are: (i) A local cell assembly level of synchronization, which is measured via the power of an oscillatory signal on EEG or MEG [^[22]^] (ii) inter-areal synchronization, which is the long range synchronization between two brain regions (e.g., the hippocampus and the rhinal cortex) measured through functional connectivity measures like lagged phase synchronization, imaginary coherence, lagged coherence etc [22].

(i) Local cell assembly synchronization: cortical inhibition hypothesis.

We fully agree with the reviewer’s comment that within the context of local cell assembly synchronization measured via Power, the primary function of the alpha oscillation is cortical inhibition [^[23]^]. A review by *Klimesch et.al.* [^[24]^] summarises that the large amplitude of alpha-oscillation represented by ERS (event related Synchronization) is associated with inhibiting of the task irrelevant areas. And the small amplitude of alpha-oscillation represented by ERD (Event related synchronization) reflects the release of inhibition. ERD represent activation of task relevant regions. Thus, alpha band power is inversely related to the cortical activity. However, other studies particularly involving internal tasks/activities, such as autobiographical memory [^[25]^], self-referential process [^[26]^], meditation [^[27]^], music listening [^[28]^], mental calculation [^[29]^], mental imagery [^[30]^] etc had shown that alpha band oscillations (power) to be directly proportional to cortical activity within the task relevant area, indexed by ERS in the corresponding areas.

(ii) Interareal alpha band synchronization: “active-processing hypothesis.”

The review by *Klimesh et.al*. [24] further investigated the nature of interareal synchronization measured through functional connectivity in the alpha band. They found increased phase synchronisation among brain areas involved in the processing of the stimuli or tasks, like during object recognition [^[31]^], encoding of spoken words [^[32]^], and even through in-depth electrodes, during face recognition memory task [^[33]^]. Studies have shown that alpha amplitude suppression is associated with a concurrent increase in inter-areal alpha-band phase synchrony during picture recognition [^[34]^], visuospatial attention [^[35]^] etc. Research involving visual working memory shows that interareal phase synchronization among executive regions and attentional to be proportional to the memory load and an index of individual’s visual working memory capacity. Internal mental task is also associated with enhanced phase synchronization [29].

Indeed, the review conducted by *Palva et.al.* [^[36]^] shows that phase synchronisation among the task relevant regions to be directly proportional to the cortical activity of the regions and thus favours “active-processing hypothesis”. Recent studies have found that robust and sustained phase synchronization of cortical oscillations among task relevant regions like frontal, parietal, motor, visual regions etc associated with high visual-spatial attention [^[37]^], motor [^[38]^], creativity [^[39]^] etc. Thus, a growing number of studies support the “active-processing hypothesis” for the alpha band interareal synchronization among the task relevant regions of the brain.

# Several other studies investigated functional brain connectivity upon listening to music. Results show a positive correlation between alpha band phase synchronization, and neuronal excitability of task relevant areas [^[40]^]. Enhanced alpha band phase synchronization was correlated with enhanced emotional, [^[41]^] perception [^[42]^] and music stimuli processing for both pleasant and unpleasant music [41]. Functional brain connectivity analysis of music supports “active-processing hypothesis” for alpha band phase synchronization.

We observed enhanced phase synchronization during sad music listening compared to sad autobiographical recall (SAR) in the alpha bands in the region of interest compromising of areas associated with emotion and memory processing. Based upon the above findings, we have interpreted enhanced phase synchronization as enhanced cortical processing/activity in the task relevant brain regions during sad music listening compared to SAR state. The interpretation is also in line with studies investigating alpha band phase synchronization during listening to music [40],[41],[42].

We did not observe a significant difference in lagged phase synchronization between music listening and baseline resting sate, as it was observed in direct comparison between the two state in earlier studies [40],[41],[42]. A prolonged sad autobiographical period of diminished phase coherence in between baseline resting state and music listening in the current study might have altered the initial state of musical intervention and may be responsible for the observed difference. Future studies with a musical stimulus of a larger duration than the SAR period are needed to ascertain it.

Research on resting state functional connectivity showed that rather than merely reflecting passive or epiphenomenal activity, it is associated with active processing of high domain cognition [^[43]^]. This is also in line with the active-processing hypothesis. A review by Stevens et.al. on resting state functional connectivity, demonstrated a positive correlation between the strength of resting state functional connectivity to behavioral and cognitive domains, including perception, language, learning and memory. We interpreted reduced brain functional connectivity (as observed during SAR state) compared to resting state as reduced cognitive processing in line with above findings.

In summary, the review conducted by *Palva et.al.* [36] concludes active-processing hypothesis for Interareal long range alpha band synchronization (functional connectivity) and alpha inhibition hypothesis for local cell assembly synchronization (ERD and ERS).

We did observe a significant enhanced current source density (Local cell assembly synchronization) during sad music listening compared to baseline resting state as well as SAR state. This represents a state of enhanced alertness and cortical processing during sad music listening. This is well in accordance with earlier studies involving music listening [^[44]^]. This further supports and strengthens the findings of interareal phase synchronization analysis.

We have added the relevant section in the manuscript accordingly at

Introduction section page 3, line 90-94.

Discussion section: page 11, line 401-411; page 12, line 424-429; page 12, line 438-444.

Minor: was the participants’ subjective assessment of mood - across the three conditions - significantly correlated with EEG alpha and gamma connectivity patterns?

We thank the reviewer for the suggestions. We looked into the correlation of mean connectivity of alpha and gamma bands across the three conditions with the subjective assessment of mood. We did not observe any significant correlation in any band or conditions. Further analysis of investigating correlation for every pair of connections in each band and condition to subjective rating also did not reveal any worth reporting results.

With best regards,
Sincerely yours,

Authors of the Manuscript.

References:

1. [] Gainotti, G. (2019). A historical review of investigations on laterality of emotions in the human brain. *Journal of the History of the Neurosciences*, *28*(1), 23-41.
2. [] Denkova E, Botzung A, Scheiber C, Manning L. Implicit emotion during recollection of past events: A nonverbal fMRI study. Brain Research. 2006;1078(1):143–150. doi:10.1016/j.brainres.2006.01.061.
3. [] Piefke M. Differential remoteness and emotional tone modulate the neural correlates of autobiographical memory. Brain. 2003;126(3):650–668. doi:10.1093/brain/awg064.
4. [] Markowitsch H, Vandekerckhove M, Lanfermann H, Russ M. Engagement of Lateral and Medial Prefrontal Areas in the Ecphory of Sad and Happy Autobiographical Memories. Cortex. 2003;39(4-5):643–665. doi:10.1016/S0010-9452(08)70858-X.
5. [] Cimino CR, Verfaellie M, Bowers D, Heilman KM. Autobiographical memory: Influence of right hemisphere damage on emotionality and specificity. Brain and Cognition. 1991;15(1):106–118. doi:10.1016/0278-2626(91)90019-5.
6. [] van den Tol, A. J. (2016). The appeal of sad music: A brief overview of current directions in research on motivations for listening to sad music. *The Arts in Psychotherapy*, *49*, 44-49.
7. [] Goshvarpour, A., & Goshvarpour, A. (2019). EEG spectral powers and source localization in depressing, sad, and fun music videos focusing on gender differences. *Cognitive neurodynamics*, *13*(2), 161-173.
8. [] Menon, V. (2015). Salience network. Brain mapping: an encyclopedic reference.
9. [] Seeley, W. W. (2019). The salience network: a neural system for perceiving and responding to homeostatic demands. *Journal of Neuroscience*, *39*(50), 9878-9882.
10. [] Bastos, A. M., & Schoffelen, J. M. (2016). A tutorial review of functional connectivity analysis methods and their interpretational pitfalls. *Frontiers in systems neuroscience*, *9*, 175.
11. [] Pascual-Marqui, R. D., Lehmann, D., Koukkou, M., Kochi, K., Anderer, P., Saletu, B., ... & Kinoshita, T. (2011). Assessing interactions in the brain with exact low-resolution electromagnetic tomography. *Philosophical Transactions of the Royal Society A: Mathematical, Physical and Engineering Sciences*, *369*(1952), 3768-3784.
12. [] Chaumon, M., Bishop, D. V., & Busch, N. A. (2015). A practical guide to the selection of independent components of the electroencephalogram for artifact correction. *Journal of neuroscience methods*, *250*, 47-63.
13. [] Crespo-Garcia, M., Atienza, M., & Cantero, J. L. (2008). Muscle artifact removal from human sleep EEG by using independent component analysis. *Annals of biomedical engineering*, *36*(3), 467-475.
14. [] Nolan, H., Whelan, R., & Reilly, R. B. (2010). FASTER: fully automated statistical thresholding for EEG artifact rejection. *Journal of neuroscience methods*, *192*(1), 152-162.
15. [] Mognon, A., Jovicich, J., Bruzzone, L., & Buiatti, M. (2011). ADJUST: An automatic EEG artifact detector based on the joint use of spatial and temporal features. *Psychophysiology*, *48*(2), 229-240.
16. [] Sburlea, A. I., Wilding, M., & Müller-Putz, G. R. (2021). Disentangling human grasping type from the object's intrinsic properties using low-frequency EEG signals. *Neuroimage: Reports*, *1*(2), 100012.
17. [] Khosravani, S., Mahnan, A., Yeh, I. L., Watson, P. J., Zhang, Y., Goding, G., & Konczak, J. (2019). Atypical somatosensory-motor cortical response during vowel vocalization in spasmodic dysphonia. *Clinical Neurophysiology*, *130*(6), 1033-1040.
18. [] Delorme, A., and Makeig, S. (2004). EEGLAB: an open source toolbox for analysis of single-trial EEG dynamics including independent component analysis. *J. Neurosci. Methods* 134, 9–21. doi: 10.1016/j.jneumeth.2003.10.009
19. [] Pascual-Marqui, R. D., Lehmann, D., Koukkou, M., Kochi, K., Anderer, P., Saletu, B., ... & Kinoshita, T. (2011). Assessing interactions in the brain with exact low-resolution electromagnetic tomography. *Philosophical Transactions of the Royal Society A: Mathematical, Physical and Engineering Sciences*, *369*(1952), 3768-3784.
20. [] Holmes, A. P., Blair, R. C., Watson, J. D. G., & Ford, I. (1996). Nonparametric analysis of statistic images from functional mapping experiments. *Journal of Cerebral Blood Flow & Metabolism*, *16*(1), 7-22.
21. [] Pascual-Marqui, R. D., Lehmann, D., Koenig, T., Kochi, K., Merlo, M. C., Hell, D., & Koukkou, M. (1999). Low resolution brain electromagnetic tomography (LORETA) functional imaging in acute, neuroleptic-naive, first-episode, productive schizophrenia. *Psychiatry Research: Neuroimaging*, *90*(3), 169-179.
22. [] Palva, S., & Palva, J. M. (2011). Functional roles of alpha-band phase synchronization in local and large-scale cortical networks. *Frontiers in psychology*, *2*, 204.
23. [] Klimesch, W. (2012). Alpha-band oscillations, attention, and controlled access to stored information. Trends in Cognitive Sciences 16, 606-617
24. [] Klimesch, W., Sauseng, P., & Hanslmayr, S. (2007). EEG alpha oscillations: the inhibition–timing hypothesis. *Brain research reviews*, *53*(1), 63-88.
25. [] Knyazev, G. G., Savostyanov, A. N., Bocharov, A. V., Dorosheva, E. A., Tamozhnikov, S. S., & Saprigyn, A. E. (2015). Oscillatory correlates of autobiographical memory. *International Journal of Psychophysiology*, *95*(3), 322-332.
26. [] Knyazev, G. G. (2013). EEG correlates of self-referential processing. *Frontiers in human neuroscience*, *7*, 264.
27. [] Aftanas, L. I., & Golocheikine, E. S. (2001). Human anterior and frontal midline theta and lower alpha reflect emotionally positive state and internalized attention: high-resolution EEG investigation of meditation. *Neuroscience letters*, *310*(1), 57-60.
28. [] Markovic A, K ̈uhnis J, J ̈ancke L. Task context influences brain activation during
    music listening. Frontiers in human neuroscience. 2017;11:342.
29. [] Palva, J. M., Palva, S., & Kaila, K. (2005). Phase synchrony among neuronal oscillations in the human cortex. *Journal of Neuroscience*, *25*(15), 3962-3972.
30. [] Schaefer, R. S., Vlek, R. J., & Desain, P. (2011). Music perception and imagery in EEG: Alpha band effects of task and stimulus. *International Journal of Psychophysiology*, *82*(3), 254-259.
31. [] Mima, T., Oluwatimilehin, T., Hiroak, T., Hallet, M., 2001. Transient interhemispheric neural synchrony correlates with object recognition. J. Neurosci. 21, 3942–3948.
32. [] Schack, B., Weiss, S., Rappelsberger, P., 2003. Cerebral information transfer during word processing: where and when does it occur and how fast is it? Hum. Brain Mapp. 19, 18–36.
33. [] Klopp, J., Marinkovic, K., Chauvel, P., Nenov, V., Halgren, E., 2000. Early widespread cortical distribution of coherent fusiform face selective activity. Hum. Brain Mapp. 11, 286–293.
34. [] Freunberger, R., Klimesch, W., Griesmayr, B., Sauseng, P., and Gruber, W. (2008). Alpha phase coupling reflects object recognition. *Neuroimage* 42, 928–935.
35. [] Doesburg, S. M., Green, J. J., McDonald, J. J., and Ward, L. M. (2009). From local inhibition to long-range integration: a functional dissociation of alpha-band synchronization across cortical scales in visuospatial attention. *Brain Res.* 1303, 97–110.
36. [] Palva, S., & Palva, J. M. (2011). Functional roles of alpha-band phase synchronization in local and large-scale cortical networks. *Frontiers in psychology*, *2*, 204.
37. [] Lobier, M., Palva, J. M., & Palva, S. (2018). High-alpha band synchronization across frontal, parietal and visual cortex mediates behavioral and neuronal effects of visuospatial attention. *Neuroimage*, *165*, 222-237.
38. [] Zazio, A., Miniussi, C., & Bortoletto, M. (2021). Alpha-band cortico-cortical phase synchronization is associated with effective connectivity in the motor network. *Clinical Neurophysiology*, *132*(10), 2473-2480.
39. [] Nobukawa, S., Yamanishi, T., Ueno, K., Mizukami, K., Nishimura, H., & Takahashi, T. (2020). High phase synchronization in alpha band activity in older subjects with high creativity. *Frontiers in human neuroscience*, *14*, 583049.
40. [] Wu, J., Zhang, J., Liu, C., Liu, D., Ding, X., & Zhou, C. (2012). Graph theoretical analysis of EEG functional connectivity during music perception. *Brain research*, *1483*, 71-81.
41. [] Flores-Gutiérrez, E. O., Díaz, J. L., Barrios, F. A., Guevara, M. Á., del Río-Portilla, Y., Corsi-Cabrera, M., & del Flores-Gutiérrez, E. O. (2009). Differential alpha coherence hemispheric patterns in men and women during pleasant and unpleasant musical emotions. *International Journal of Psychophysiology*, *71*(1), 43-49.
42. [] Flores-Gutiérrez, E. O., Díaz, J. L., Barrios, F. A., Favila-Humara, R., Guevara, M. Á., del Río-Portilla, Y., & Corsi-Cabrera, M. (2007). Metabolic and electric brain patterns during pleasant and unpleasant emotions induced by music masterpieces. *International Journal of Psychophysiology*, *65*(1), 69-84.
43. [] Stevens WD, Spreng RN. Resting-state functional connectivity MRI reveals active processes central to cognition. Wiley Interdisciplinary Reviews: Cognitive Science. 2014;5(2):233–245.
44. [] Jäncke, L., Kühnis, J., Rogenmoser, L., & Elmer, S. (2015). Time course of EEG oscillations during repeated listening of a well-known aria. *Frontiers in human neuroscience*, *9*, 401.
